# Examining the Impact of Local Constraint Violations on Energy Computations in DFT

**DOI:** 10.1002/jcc.70005

**Published:** 2025-01-02

**Authors:** Vaibhav Khanna, Bikash Kanungo, Vikram Gavini, Ambuj Tewari, Paul M. Zimmerman

**Affiliations:** ^1^ Department of Chemistry University of Michigan Ann Arbor Michigan USA; ^2^ Department of Mechanical Engineering University of Michigan Ann Arbor Michigan USA; ^3^ Department of Materials Science & Engineering University of Michigan Ann Arbor Michigan USA; ^4^ Department of Statistics University of Michigan Ann Arbor Michigan USA

**Keywords:** exact conditions, exchange‐correlation (XC) functionals, extent of violation index (EVI), local constraints density functional theory (DFT)

## Abstract

This work examines the impact of locally imposed constraints in Density Functional Theory (DFT). Using a metric referred to as the extent of violation index (EVI), we quantify how well exchange‐correlation functionals adhere to local constraints. Applying EVIs to a diverse set of molecules for GGA functionals reveals constraint violations, particularly for semi‐empirical functionals. We leverage EVIs to explore potential connections between these violations and errors in chemical properties. While no correlation is observed for atomization energies, a significant statistical correlation emerges between EVIs and total energies. Similarly, the analysis of reaction energies suggests weak positive correlations for specific constraints. However, definitive conclusions about error cancellation mechanisms cannot be made at this time. These observations revealed by EVIs may be useful for consideration when designing future generations of semilocal functionals.

## Graphical abstract

Local constraint violations by the BP86 functional, quantified by our metric, trending with total energy errors: molecules with more violations also have higher errors in DFT total energies.

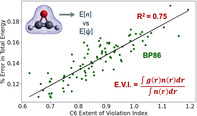

## 1 Introduction

Density Functional Theory (DFT) has become an indispensable tool used extensively by chemists, physicists and material scientists [1]. Under the Kohn‐Sham ansatz [2] a set of non‐interacting single‐particle states are generated to represent the electron density. Most components of the DFT energy are known, except for the exchange‐correlation term $E_{\mathrm{xc}}$.

(1) $$\begin{array}{c} E\left[n\left(r\right)\right]=T_{s}+\frac{1}{2}\int \int \frac{n\left(r_{1}\right)n\left(r_{2}\right)}{|r_{1}-r_{2}|}dr_{1}dr_{2}\\ \;-\sum_{k}^{\text{nuclei}}\int \frac{Z_{k}}{|r-R_{k}|}n\left(r\right)dr+E_{\mathrm{xc}}\left[n\left(r\right)\right] \end{array}$$

(2) $$T_{s}=-\frac{1}{2}\sum_{i}^{N}\left\langle \phi_{i}|\nabla_{i}^{2}|\phi_{i}\right\rangle$$

(3) $$n\left(r\right)=\sum_{i=1}^{N}{\left|\phi_{i}\left(r\right)\right|}^{2}$$

The term $E_{\mathrm{xc}}\left[n\left(r\right)\right]$ in Equation (1), referred to as the exchange‐correlation functional, encompasses the corrections to the kinetic energy that arise due to the interacting nature of electrons and all non‐classical components of the Coulomb energy. Even though the exact form of this functional remains unknown, several approximations have been developed over time that has given rise to a range of exchange‐correlation functionals [3].

While the exact exchange‐correlation functional remains elusive, its analytical properties have guided and continue to guide functional development [4, 5, 6, 7]. These analytical properties are referred to as exact conditions. Functionals that were constructed to satisfy a number of these exact conditions are generally referred to as non‐empirical, for instance SCAN [8], PW91 [9, 10] and PBE [11]. These functionals are among the best choices for solid state DFT calculations [12]. Recently, Pederson and Burke [13] provided local expressions for six exact conditions, then evaluated these across a range of densities. They found that for some densities, semi‐empirical functionals (to be discussed below) violated local constraints, and showed non‐empirical functionals satisfied the constraints within a numerical threshold.

Table 1 lists the six exact conditions along with their local versions. Each condition is a mathematically precise statement that must be satisfied by the exact functional. The corresponding local constraints listed in the table are excessive; satisfying these constraints guarantees that the corresponding exact condition is satisfied, but violating them does not necessarily imply violations to exact conditions [14, 15, 16, 17, 18, 19]. The first condition of Table 1 states that the DFT correlation energy should be non‐positive, a concept consistent with the stabilizing effect of correlation. Since electron correlation reduces Coulomb repulsion among electrons, nonpositivity of $E_{c}$ is reasonable. While enforcing the local version of this condition ($\varepsilon_{c}\left[n\right]\left(r\right)\leq 0$) is not strictly necessary, it can be useful in the context of semi‐local functionals and guarantees the global condition is met. Conditions 3 and 4 are derived by stretching the electron density in space, where the exact density functional would display specific scaling inequalities in the correlation energy $E_{c}$ and the kinetic contribution to the correlation energy $T_{c}$ [16, 17, 18]. Additional conditions coming from upper and lower bounds on the exchange‐correlation potential energy ($U_{\mathrm{xc}}$) can also be applied [14]. Lower bounds have been derived from the uniform electron gas, which underpin conditions 2 and 5. These conditions are typically referred to as Lieb‐Oxford bounds, and involve the Lieb‐Oxford constant, $C_{\mathrm{LO}}$, whose value is taken as 2.27 [13]. Condition 6 is closely related to the adiabatic connection [20] where the exchange‐correlation energy is given as: $E_{\mathrm{xc}}=\int_{0}^{1}U_{\mathrm{xc}}^{\lambda }\mathrm{d\lambda}$. In this expression, λ is the coupling constant, which goes from 0 to 1, reflecting a transition from a system of non‐interacting electrons to a system of interacting electrons. $U_{\mathrm{xc}}^{\lambda }$ is the change in exchange‐correlation energy with respect to the coupling strength λ. This formula effectively links the non‐interacting Kohn‐Sham reference system with the fully interacting system through a sequence of partially interacting systems, all of which share the same density. Focusing only on the correlation contribution, as λ increases, it is imperative that the correlation energy decreases. This gives rise to the monotonicity condition for $U_{c}$, which reduces the occurrence of unphysical behaviors in the correlation energy [19].

**TABLE 1:** Exact conditions in DFT and their local counterparts. Locally imposed constraints involve quantities such as the correlation energy density $\varepsilon_{c}\left[n\right]\left(r\right)$, exchange (correlation) enhancement factor, defined as $F_{x\left(c\right)}$ = $\varepsilon_{x\left(c\right)}\left[n\right]\left(r\right)$/$\varepsilon_{x}^{\text{unif}}\left[n\right]\left(r\right)$ where $\varepsilon_{x}^{\text{unif}}$ is the exchange energy density for an unpolarized uniform electron gas (given as $\varepsilon_{x}^{\text{unif}}$ = $-\left(3/4\pi \right){\left(3\pi^{2}n\right)}^{1/3}$), the Wigner‐Seitz radius computed as $r_{s}$ = ${\left(4\mathrm{\pi n}/3\right)}^{-1/3}$ and the Lieb‐Oxford constant $C_{\mathrm{LO}}$, taken to be equal to 2.27 [13].

#	Condition name	Exact condition	Local constraint
1	$E_{c}$ non‐positivity [13]	$E_{c}\left[n\right]\leq 0$	$\varepsilon_{c}\left[n\right]\left(r\right)\leq 0$
2	$E_{\mathrm{xc}}$ lower bound [13, 14, 15]	$E_{\mathrm{xc}}\left[n\right]\geq C_{\mathrm{LO}}\int dr\;n\left(r\right)\varepsilon_{x}^{\text{unif}}\left[n\right]\left(r\right)$	$F_{\mathrm{xc}}\leq C_{\mathrm{LO}}$
3	$E_{c}$ scaling inequality [13, 16]	$\left(\gamma -1\right)E_{c}\left[n_{\gamma }\right]\geq \gamma \left(\gamma -1\right)E_{c}\left[n\right]$	$\frac{\partial F_{c}}{\partial r_{s}}\geq 0$
4	$T_{c}$ upper bound [13, 17, 18]	$T_{c}\left[n_{\gamma }\right]\leq -\gamma \left({\left.\frac{\partial E_{c}\left[n_{\gamma }\right]}{\partial \gamma }\right|}_{\gamma \to 0}\right)+E_{c}\left[n_{\gamma }\right]$	$\frac{\partial F_{c}}{\partial r_{s}}\leq \frac{F_{c}\left(r_{s}\to \infty \right)-F_{c}}{r_{s}}$
5	$U_{\mathrm{xc}}$ lower bound [13, 14]	$U_{\mathrm{xc}}\left[n\right]\geq C_{\mathrm{LO}}\int dr\;n\left(r\right)\varepsilon_{x}^{\text{unif}}\left[n\right]\left(r\right)$	$F_{\mathrm{xc}}+r_{s}\frac{\partial F_{c}}{\partial r_{s}}\leq C_{\mathrm{LO}}$
6	$U_{c}\left(\lambda \right)$ monotonicity from	$\frac{dU_{c}\left(\lambda \right)}{d\lambda }\leq 0$	$\frac{\partial }{\partial r_{s}}\left(r_{s}^{2}\frac{\partial F_{c}}{\partial r_{s}}\right)\geq 0$
	Adiabatic connection [13, 19]		

An alternative category of functionals, commonly referred to as semi‐empirical, holds a significant position in molecular DFT methods. As the name suggests, these functionals are inherently parameterized by fitting to benchmark datasets. Functionals such as BLYP [21, 22, 23], BP86 [21, 24], M06‐L [25, 26], and B3LYP [27] fall in this category. These semi‐empirical functionals are designed to capture the chemical properties of molecular systems by best fits to benchmark results from accurate wavefunction computations [3]. However, it is important to recognize that even with a sophisticated functional form and access to extensive training data, these functionals may face challenges when applied to systems that lie outside their original training domain, such as solids [13, 28, 29, 30]. This highlights the ongoing need to create functionals that not only excel within their training domains but also exhibit enhanced generalization capabilities to handle a wider array of chemical species and materials. While modern semi‐empirical functionals, such as M11, include transition metal data in their training sets, accurate modeling of transition metals, and more broadly, periodic systems and materials, still remains challenging for both semi‐empirical and non‐empirical functionals [31, 32, 33, 34, 35, 36, 37, 38, 39, 40, 41, 42].

An exact exchange‐correlation functional is anticipated to satisfy all exact conditions, including but not limited to those present in Table 1. However, it is important to recognize that such a functional, while adhering to the global exact conditions, could still violate local constraints in specific regions. In light of this, and the continued success of semi‐empirical functionals [3, 35, 43, 44, 45], one might ask: is there any correlation between satisfying local constraints and predicting chemical properties?

The above question might be addressed, at least to a certain degree, through metrics that measure the degree of violation of local constraints. An exact functional would adhere precisely to all known exact conditions, and it is possible that the degree of violation of exact conditions would correlate with errors in property evaluation. No present‐day functional is anywhere near exact, so it is unclear whether the same assumption applies to contemporary functionals and locally imposed constraints. This leads us to analyze contemporary approximate density functionals and seek relationships between local constraints and energetic properties. The methods section introduces an index that measures the extent of violation, averaged over a molecule's electron density, which may be useful in finding statistical correlations between local constraints and electronic energies.

**FIGURE 1:**
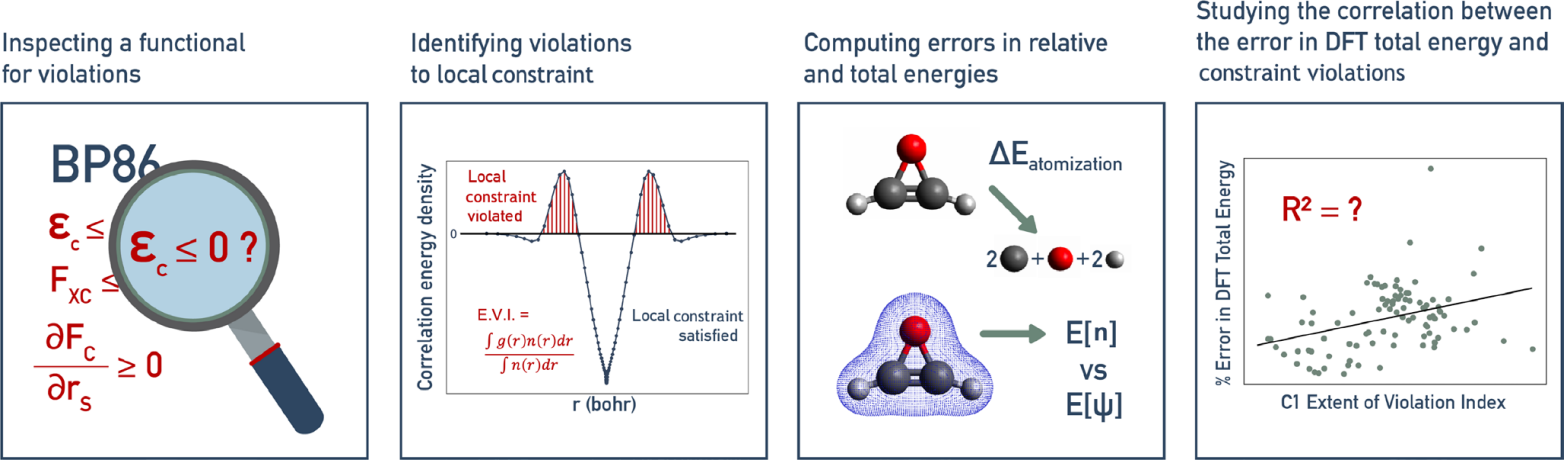
Local constraint analysis workflow: Close inspection of a functional reveals violation of a local constraint. Errors in atomization and DFT total energies are calculated, and the relationship between the error in DFT total energy and constraint violations is explored.

This study examines a selection of non‐empirical and semi‐empirical generalized gradient approximation (GGA) exchange‐correlation functionals. Our investigation assesses these functionals for potential deviations from local constraints for a diverse range of molecules (Figure 1). Furthermore, local constraints corresponding to conditions 1, 3, 4, and 6 apply specifically to correlation functionals. Thus, hybrid functionals such as B3LYP, B3P86, and PBE0 [22, 23, 24, 27, 46] are represented in this study through their GGA correlation components. Errors in total and relative energies predicted by these functionals are related to the extent to which a functional adheres to these constraints, demonstrating that the new violation index is a useful means for examining approximate semi‐local density functionals.

## 2 Methods

In order to investigate the chemical significance of local constraints, we studied the impact of violating these constraints on predictions of two chemical properties, namely atomization and reaction energies. Reliable values of these properties were obtained from two databases, namely W4‐11 and G2RC, which are often used in DFT benchmark studies [47, 48, 49]. We restricted our study to GGA density functionals, since they form the simplest semi‐local models and are used extensively. In order to compare the extent to which different functionals violate a given condition, we quantified violations by computing the extent of violation index, which is defined later in this section.

**FIGURE 2:**
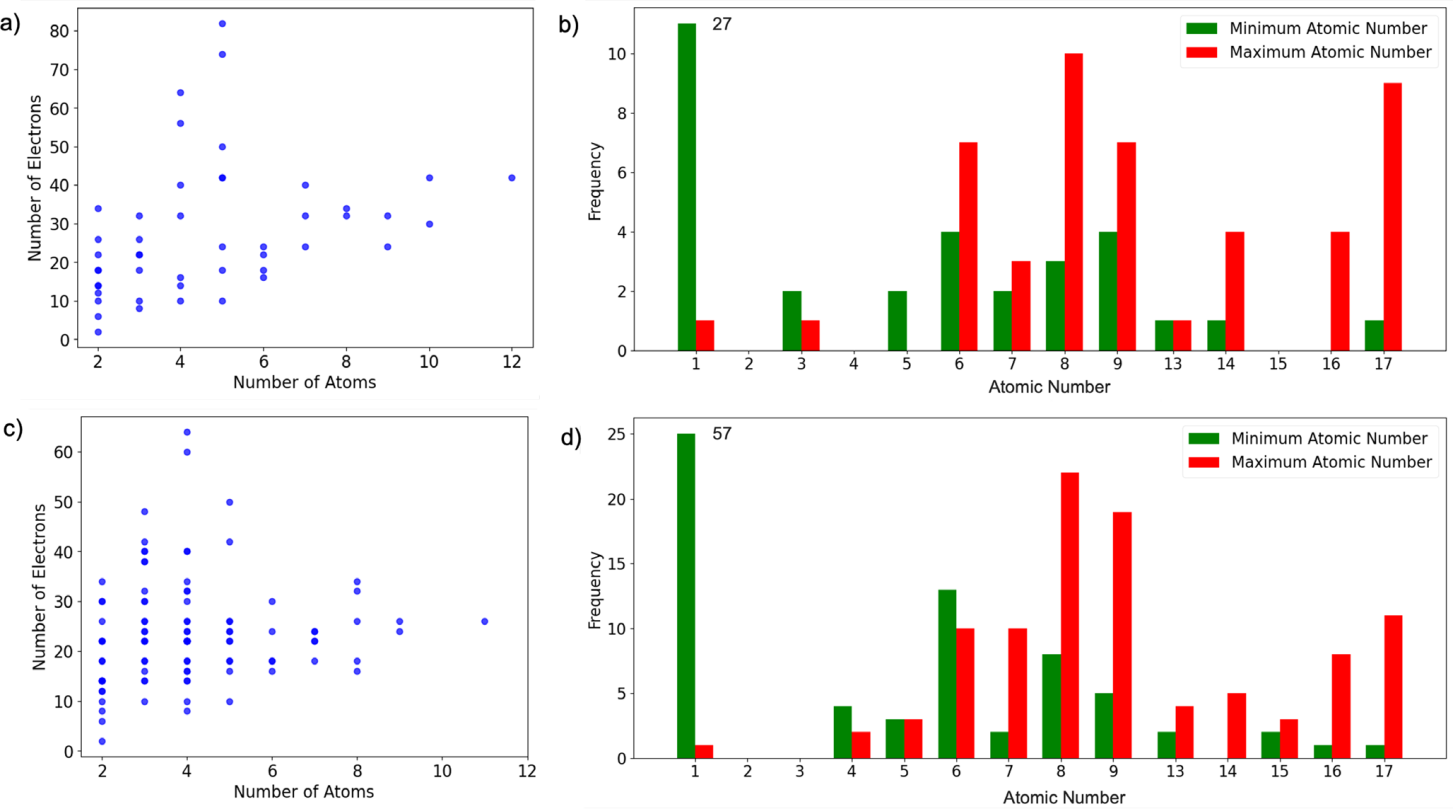
Plots summarizing the properties of molecules in the G2RC (a and b) and W4‐11 (c and d) databases. The frequency values have been clipped to 11 and 25 in plots b and d.

In this study, we selected closed‐shell neutral molecules from the W4‐11 and G2RC databases. This selection was prompted by the fact that closed‐shell neutral molecules exhibit simplified electronic structures, avoiding strong correlations where DFT is generally less precise. Figure 2 delineates chemical space that is represented in the two datasets. The W4‐11 database contains a diverse range of 140 molecules, from diatomics to medium‐sized organic compounds. These molecules predominantly consist of atoms from the second row of the periodic table, although some also incorporate atoms from the third row, and a few have a combination of both. On the other hand, the G2RC database, while similar in elemental composition, features a smaller number of molecules, providing reaction energies for 25 reactions.

To generate electron densities for the selected molecules, we employed very dense (PySCF level 9) Becke atomic grids (200 radial and 1454 angular grid points) [50] using the PySCF program [51, 52]. All computations used the augmented, polarized, triple‐zeta basis set, aug‐cc‐pVTZ [53].

The functionals employed in this work have been broadly categorized into two classes: non‐empirical and semi‐empirical GGAs. Non‐empirical functionals (PBE, PW91, AM05 [54, 55]) prioritize rigorous adherence to exact conditions, with no fitting to molecular properties. Conversely, semi‐empirical functionals (BLYP, BP86, OLYP [22, 23, 56], SOGGA11 [57], GAM [58], N12 [59]) incorporate varying degrees of empirical parameter fitting to target desired chemical accuracy. Not all functionals in the empirical category are equally empirical, however. BLYP, with only two parameters fitted to experiment (in the LYP part), resembles non‐empirical functionals like PBE, and is distinct from functionals with extensive fitting like SOGGA11 (which also satisfies the second‐order density‐gradient constraint within a generalized gradient approximation). It should be kept in mind, therefore, that the classifier “empirical” can refer to functionals that were trained very differently from one another.

We employed PySCF to calculate errors in DFT total energies of molecules, using the CCSD(T) method as the ground truth. Expressions for both semi‐empirical and non‐empirical GGA functionals (listed above) and their derivatives with respect to the density were obtained from the LibXC library [60]. Defining the exchange‐correlation energy ($E_{\mathrm{xc}}$) solely via the energy density is not well‐posed in DFT, since one can always add a gauge to the energy density that integrates to zero and leaves the energy unchanged. Standard functionals in LibXC, however, have a fixed form for the energy density. Therefore, the extent of violation index was evaluated using the gauge provided by standard functionals in LibXC. All source code for the calculations described in this work can be found on our group's GitHub page at https://github.com/ZimmermanGroup/Local_Conditions_DFT.

In order to evaluate local constraints, the correlation energy density $\varepsilon_{c}\left[n\right]\left(r\right)$ for each functional was obtained from the LibXC library [60]. The exchange‐correlation enhancement factor was computed as $F_{\mathrm{xc}}$ = $\varepsilon_{\mathrm{xc}}\left[n\right]\left(r\right)/\varepsilon_{x}^{\text{unif}}\left[n\right]\left(r\right)$ where $\varepsilon_{x}^{\text{unif}}$ is the exchange energy density for an unpolarized uniform electron gas and is given as $\varepsilon_{x}^{\text{unif}}$ = $-\left(3/4\pi \right){\left(3\pi^{2}n\right)}^{1/3}$. The Wigner‐Seitz radius was computed as $r_{s}$ = ${\left(4\mathrm{\pi n}/3\right)}^{-1/3}$. The derivative $\partial F_{c}/\partial r_{s}$ was calculated by substituting $F_{c}$ as $\varepsilon_{c}\left[n\right]\left(r\right)$/$\varepsilon_{x}^{\text{unif}}\left[n\right]\left(r\right)$ and making use of the quotient rule.

### 2.1 Extent of Violation Index

Each local constraint was evaluated across the entire grid for each molecule, establishing a distribution of values, $g\left(r\right)$, which represents the deviation from that local constraint. This information is condensed into a metric to quantify the overall degree in which the local constraint is violated. This work therefore defines the Extent of Violation Index (EVI) as

(4) $$\mathrm{EVI}=\frac{\int g\left(r\right)n\left(r\right)dr}{\int n\left(r\right)dr}$$

(5) $$\begin{array}{c} g\left(r\right)=\left\{\begin{array}{ll} \left|\text{violation}\right| & \text{if local constraint is violated}\\ 0 & \text{otherwise} \end{array}\right. \end{array}$$

For example, while evaluating the second local constraint, if at a grid point, $F_{\mathrm{xc}}>C_{\mathrm{LO}}$, $g\left(r\right)$ will be equal to $F_{\mathrm{xc}}-C_{\mathrm{LO}}$. If, however, $F_{\mathrm{xc}}\leq C_{\mathrm{LO}}$, $g\left(r\right)$ will be set to zero. Numerical integration over the grid gives the extent of violation. While violating local constraints does not strictly imply the global exact conditions are violated, evaluation of the local constraints is nonetheless useful for assessing functionals [13].

The EVI was calculated for every molecule in the two databases and all nine GGA functionals considered in this study. We also computed EVI for reactions in the G2RC dataset. The violation index for a reaction was computed as follows. Consider the following reaction: A + B → C + D. For any local constraint $C_{m}$, we computed the EVI for this reaction as:

(6) $${\mathrm{EVI}}_{\text{reaction}}^{C_{m}}\;=\;N_{C}\;{\mathrm{EVI}}_{C}^{C_{m}}\;+\;N_{D}\;{\mathrm{EVI}}_{D}^{C_{m}}\;-\;N_{A}\;{\mathrm{EVI}}_{A}^{C_{m}}\;-\;N_{B}\;{\mathrm{EVI}}_{B}^{C_{m}}$$

Multiplication of EVIs of molecules with their number of electrons ($N_{i}$) was required since EVIs for each individual molecule were normalized.

Our violation indices differ from the metric computed by Pederson and Burke for evaluating local constraints [13]. In their work, Pederson and Burke constructed a range of electron densities and their gradients over uniform spacing between realistic limits. Relevant derivatives involved in local constraints were then computed numerically. They reported the fraction of grid points where local constraints were violated, where violations were computed using predefined tolerance values (reported in Table SII in the Supporting Information). In comparison, our metric includes the magnitude of violation as well as weighting (and averaging) by the density. While similar in spirit to the metric of Reference [13] additional concepts will be revealed by the EVI metric used herein.

## 3 Results

Nine GGA exchange‐correlation functionals were examined for their adherence to the local constraints of Table 1. First to be examined are non‐empirical functionals, which are built to satisfy a number of exact conditions. For example, the non‐empirical functional PBE by construction satisfies several energetically significant conditions, such as correlation energy non‐positivity (Condition 1 in Table 1), Lieb‐Oxford bounds (Conditions 2 and 5), $E_{c}$ scaling inequality (Condition 3), $T_{c}$ upper bound (Condition 4), uniform scaling to the high‐density limit for the correlation energy, uniform density scaling for exchange energy, the exact exchange energy spin‐scaling relationship, and the linear response of the spin‐unpolarized uniform electron gas [11]. Next, the local constraints for semi‐empirical functionals will be examined. These functionals were primarily designed to predict chemical properties by fitting to benchmark results involving molecular systems. An example of this category is the SOGGA11 functional, that has a flexible functional form which satisfies two physical constraints (the uniform electron gas limit and the second order density‐gradient expansion) and has 18 free parameters that are optimized by fitting to 15 chemical databases [57]. For each semi‐empirical functional, the relationship between local constraints and their impact on successful predictions of chemical properties will be studied.

### 3.1 Non‐empirical Functionals

**FIGURE 3:**
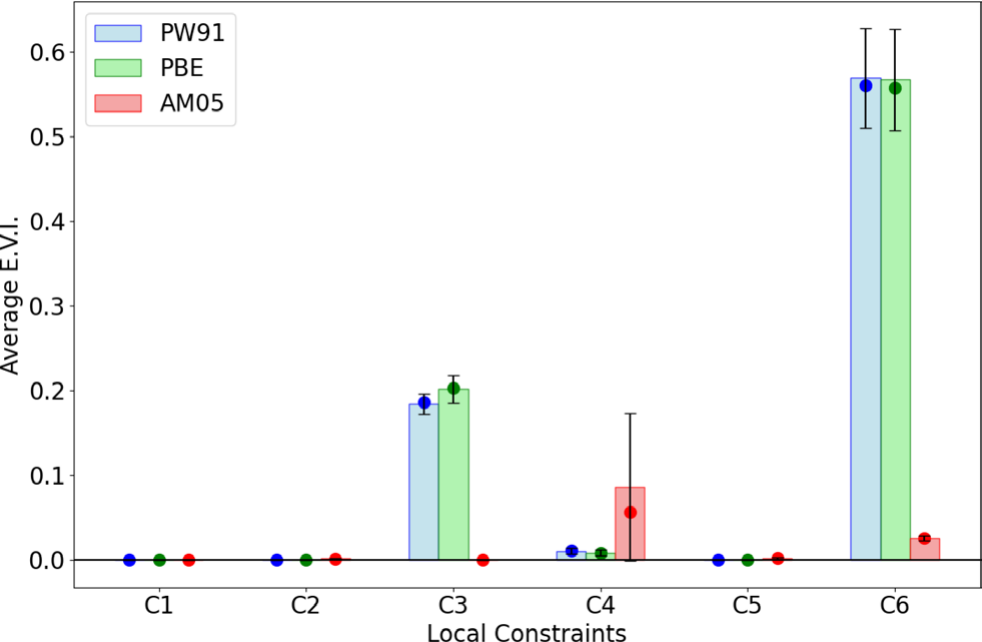
Distribution of extent of violation indices for all local constraints for non‐empirical functionals PBE, PW91 and AM05 (W4‐11 database). For each local constraint, the median value, average and standard deviation of EVIs is displayed.

First, we investigate non‐empirical GGA functionals for violations to local constraints. For the three non‐empirical functionals, namely PBE, PW91 and AM05, Figure 3 shows the distribution of EVIs for all local constraints. EVIs are computed over the set of neutral closed shell molecules in the W4‐11 database for each functional, then the distributions over these values are in Figure 3. While these functionals were constructed [9, 10, 11, 54, 55] to satisfy exact conditions 1, 2, and 5, ($E_{c}$ non‐positivity, $E_{\mathrm{xc}}$ lower bound, and $U_{\mathrm{xc}}$ lower bound local constraints) they also satisfy the corresponding locally imposed constraints, as seen by the zero EVI values.

However, the picture becomes more interesting when considering local constraints 3 and 4. These constraints relate to the scaling behavior of the correlation energy and upper bound on the kinetic contribution to the correlation energy. While PBE and AM05 are known to satisfy the corresponding exact conditions for any density [11, 13, 54, 55], we observe non‐zero EVIs for local constraints 3 and 4. Violations to constraint 6 were also observed, and this constraint will be discussed further in the next section.

### 3.2 Local Constraints and Chemical Properties

Investigation of semi‐empirical functionals (BLYP, OLYP, BP86, SOGGA11, N12, and GAM) revealed violations to all local constraints, albeit to varying extents. The average violation indices for 96 closed shell molecules in the W4‐11 database are reported in Table 2 for all local constraints.

**TABLE 2:** Average extent of violation indices for all local constraints for semi‐empirical functionals, reported for closed shell neutral molecules in the W4‐11 database.

Functional	C1 average EVI	C2 average EVI	C3 average EVI	C4 average EVI	C5 average EVI	C6 average EVI
BLYP	0.0002	0.0025	0.0855	0.0295	0.0020	1.4852
OLYP	0.0002	0.0006	0.0855	0.0295	0.0003	1.4852
BP86	0.0000	0.0029	0.1729	0.0010	0.0070	0.8739
SOGGA11	0.0000	0.0192	0.0805	0.3768	0.2288	2.5847
N12	0.0000	0.0030	0.1010	0.0705	0.1279	1.4819
GAM	0.0008	0.0000	0.0924	9.1014	0.0003	2.7285
Range	0.0000–0.0008	0.0000–0.0192	0.0805–0.1729	0.0010–9.1014	0.0003–0.2288	0.8739–2.7285

In order to better understand magnitudes of extent of violation indices, we look at the range of values of quantities that appear in local constraints. These are reported in Table SI in the Supporting Information (SI) for He and the BLYP functional. *Constraint 1*: The correlation energy density values range from −0.05 au close to the nucleus, to 0.02 away from it. The average EVI values for the $E_{c}$ non‐positivity condition in Table 2 are two orders of magnitude smaller than the most positive correlation energy density value (0.0002 vs. 0.0217). *Constraints 2 and 5*: The exchange‐correlation enhancement factor, which is the dominant term in local constraints 2 and 5, has values close to 1 in the vicinity of the He atom. Large values of the enhancement factor, that exceed the Lieb‐Oxford constant (taken to be 2.27), are seen at large distances from the nucleus, where there is little electron density. Much larger values (around 490 au) are also encountered at very large distances due to diminishing values of the denominator of the enhancement factor ($\varepsilon_{x}^{\text{unif}}$ = $-\left(3/4\pi \right){\left(3\pi^{2}n\right)}^{1/3}$, *n* → 0). *Constraint 3*: The values of $\partial F_{c}/\partial r_{s}$ range from −0.57 to 0.08. Since EVI values for all semi‐empirical functionals studied here exceed 0.08 (which is the maximum value of $\partial F_{c}/\partial r_{s}$ for He) for constraint 3, these functionals exhibit significant violations to the $E_{c}$ scaling inequality local constraint (C3). *Constraint 6*: Along similar lines, we conclude that these functionals also show large violations to the $U_{c}$ monotonicity local constraint (C6). *Constraint 4*: For the fourth constraint, discerning general trends proves to be challenging; the EVI values range from 0.0010 to 9.1014. For any given functional, we usually see at least two classes of molecules, one with EVIs close to zero and the other with larger EVIs. (Figures S12—S17).

**FIGURE 4:**
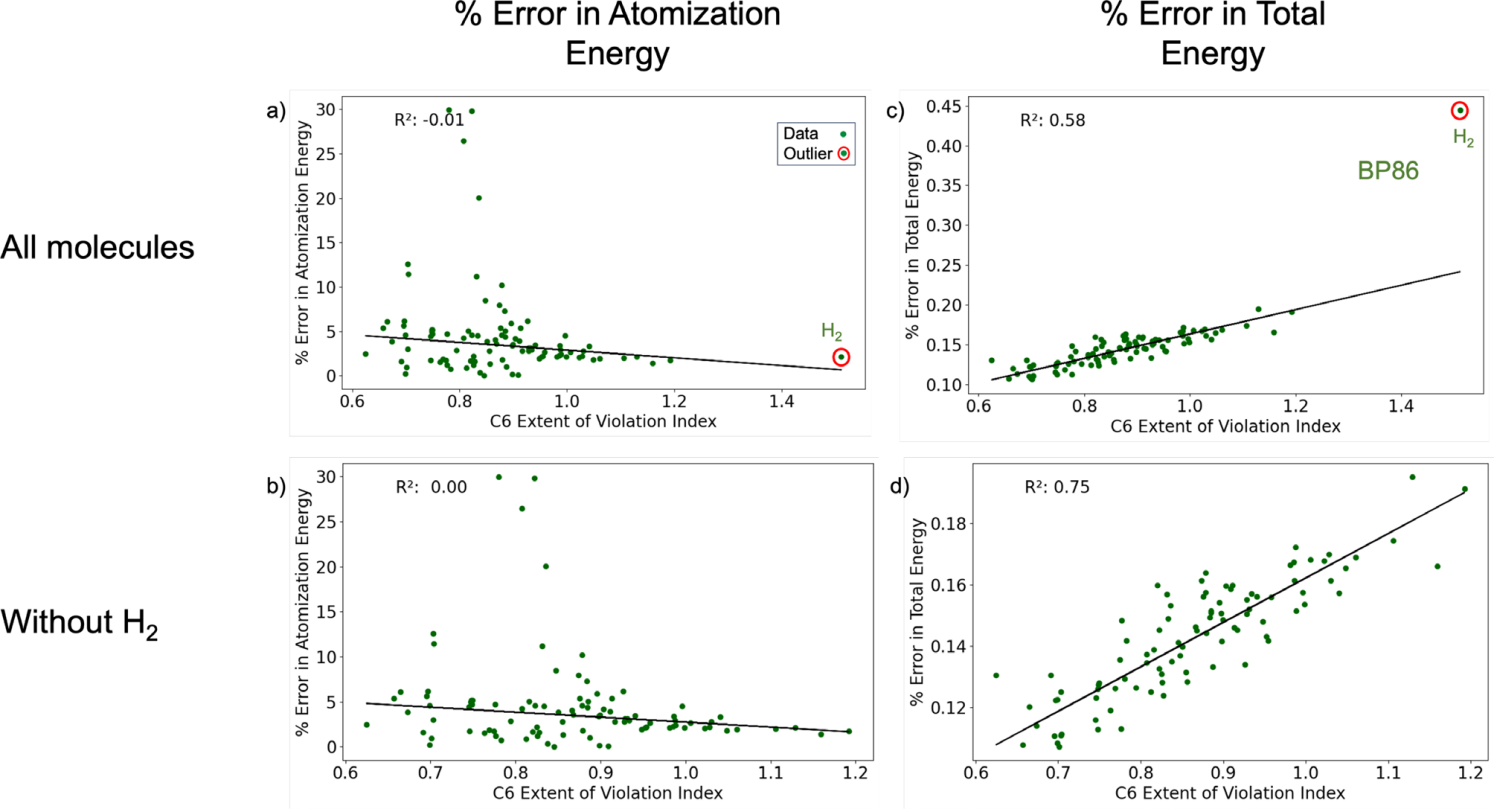
Variation of % error in atomization energy (a, b) and % error in total energy (c, d) with the extent of violation index for local constraint 6 for BP86 functional. Figures b and d exclude H_2_ (W4‐11 database).

The EVI scores of Table 2 indicate statistically notable deviations from local constraints, but do not indicate precisely how they might affect chemical properties. To understand the relationship between the extent of violation index values and chemical property predictions, we studied the correlation between these scores and the errors in atomization energies for all 96 closed‐shell neutral species in the W4‐11 database. As an example of typical results (see Figures S5, S6, S7 in SI for full results), Figure 4a shows the variation of percent error in atomization energy with the extent of violation index for the $U_{c}$ monotonicity local constraint (C6) for BP86. There appears to be no correlation between the two quantities, as evident from an $R^{2}$ value close to zero. Repeating this exercise with all other constraints (Figure S7) yields the same result: the error in atomization energy is found to be insensitive to EVI.

### 3.3 Local Constraints and DFT Total Energies

Having examined the relationship between EVI and atomization energies, the total energy was considered next. For the closed‐shell molecules studied herein, CCSD(T) provides excellent total energies as benchmark values [61, 62]. Hence, we computed the difference between total energies predicted by semi‐empirical functionals and CCSD(T) total energies. We consider this difference as the error in DFT total energy, and study its correlation with the EVI for constraints with significant violations. Figure 4c shows the variation of percent error in total energy with the extent of violation index for the $U_{c}$ monotonicity local constraint (C6) and the BP86 functional for molecules in the W4‐11 database. Significant correlation between the two quantities was indicated by the $R^{2}$ value of 0.58.

**FIGURE 5:**
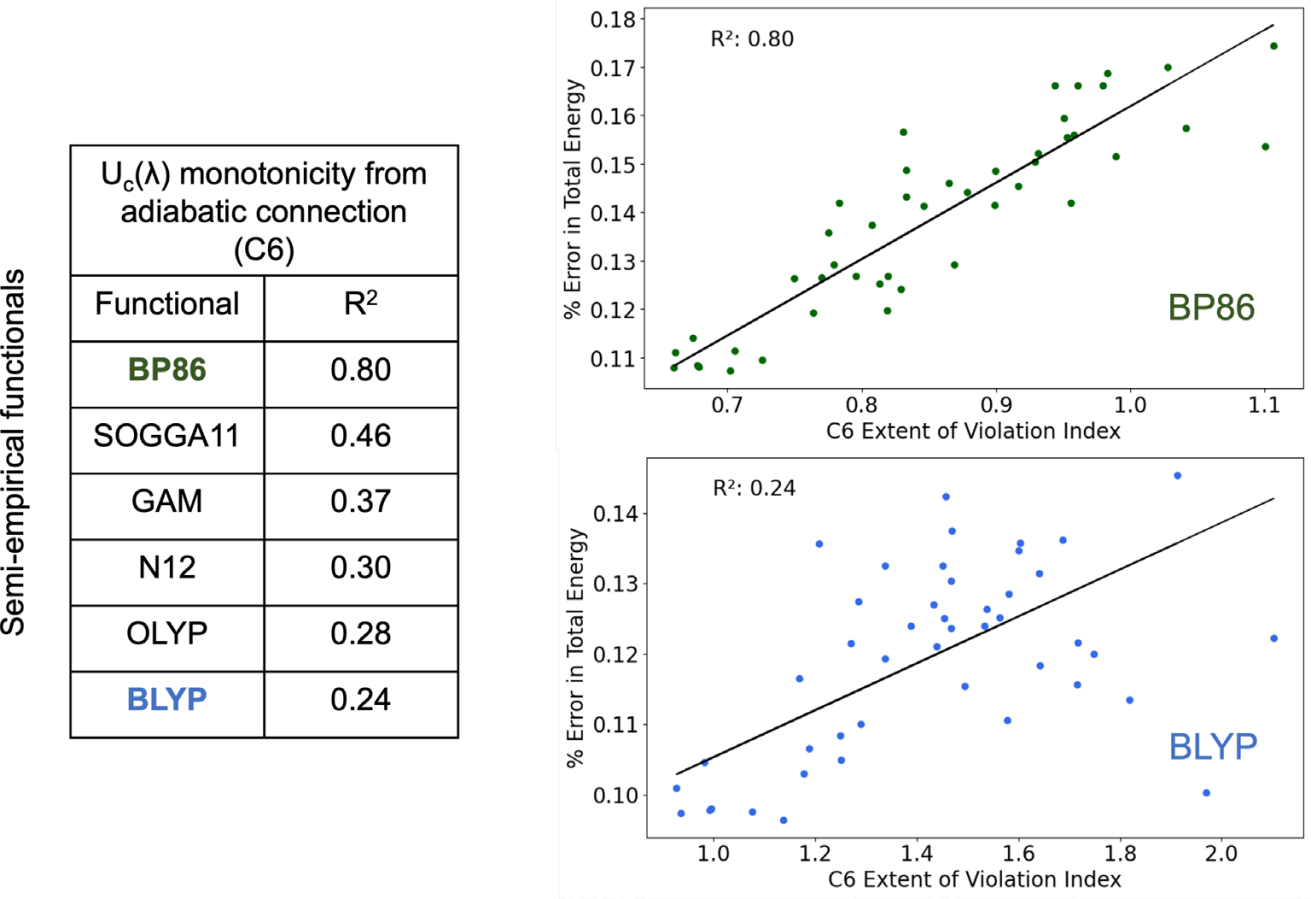
*R* ^2^ values from the variation of percent error in total energy with the extent of violation index plots for local constraint 6 for semi‐empirical functionals reported for the G2RC database (excluding outliers). Shown are the BP86 and BLYP functionals, which have the highest and lowest *R* ^2^ values.

The other semi‐empirical functionals considered in this study also showed the same trend, the errors in their total energy predictions correlated with their violation indices for constraint 6. However, the extent of this correlation varied across different functionals. Figure 5 shows the percent error in total energy vs. C6 violation index for BP86 and BLYP functionals. These plots focus on the closed‐shell neutral molecules that appear in the 25 reactions in the G2RC database. BP86 shows an $R^{2}$ value of 0.80, and BLYP displaying a weaker trend with $R^{2}$ of 0.24. The figure also presents the $R^{2}$ values for other functionals, which exhibit substantial variation. This variation is expected, considering that these GGA functionals were constructed in different ways, involving varying levels of data fitting: ranging from 2 empirical parameters in BLYP to 18 parameters fit to 15 chemical datasets in SOGGA11.

In both the atomization energy and total energy plots, we saw that molecules $H_{2}$ and ${\mathrm{Be}}_{2}$ were outliers (the latter is shown in Figure S3 in the SI). These outliers persisted in similar analyses of constraints other than Constraint 6 (Figure S7 in SI). Consequently, we recalculated the $R^{2}$ values without considering these outliers, as shown in Figure 4 (b) and (d). While no correlation with atomization energy was found without the outliers, the correlation between violation index and error in total energy strengthened. $H_{2}$ was the only two‐electron system considered in our study, which could explain its distinct behavior, and ${\mathrm{Be}}_{2}$ is unique as a near‐zero bond order system. Hence, commonly used density functional approximations fail to give accurate predictions for either of these two chemical species [63].

**FIGURE 6:**
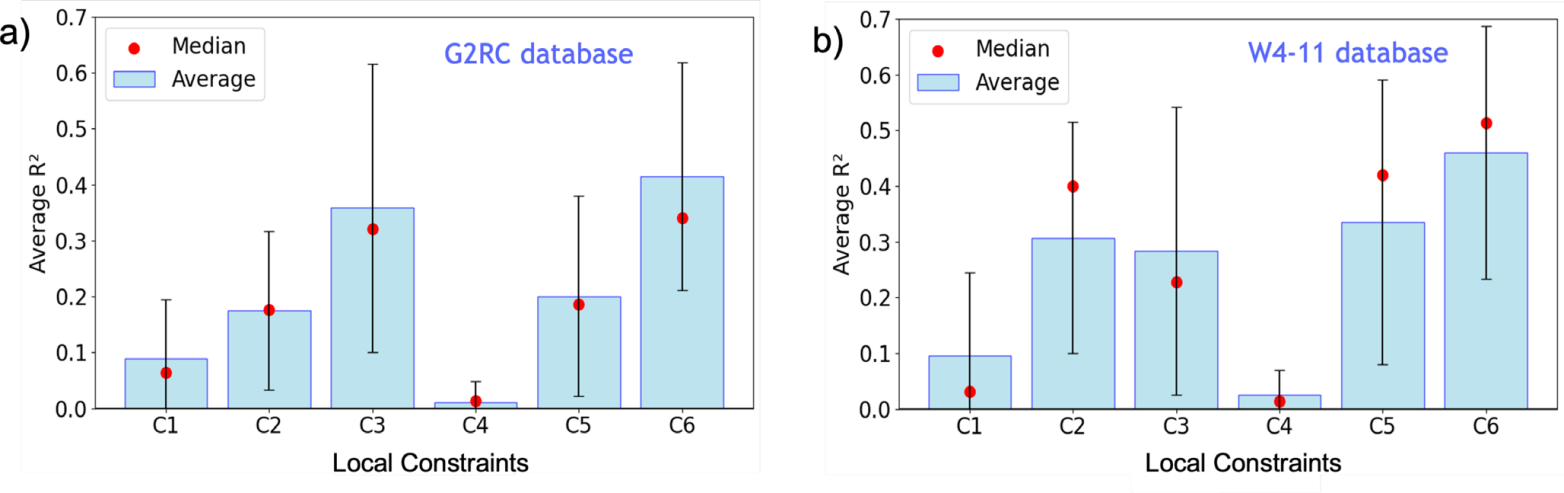
Average values of *R* ^2^ relating EVI to total energy for semi‐empirical functionals across (a) G2RC and (b) W4‐11 databases.

So far, only violation indices for the $U_{c}$ monotonicity local constraint have been examined. Statistical correlations between errors in total energies and EVI are also observed for other local constraints. Figure 6 shows the average $R^{2}$ values for all local constraints. Notably, not every constraint significantly correlates with the total energy. The $R^{2}$ value for the $T_{c}$ upper bound local constraint (C4) is nearly zero. The $E_{c}$ scaling inequality (C3) and the $U_{c}$ monotonicity local constraints (C6) have the largest $R^{2}$ overall across the two benchmark sets. The $R^{2}$ values for $E_{\mathrm{xc}}$ and $U_{\mathrm{xc}}$ lower bound local constraints (C2 and C5), however, are nearly as large as those of C3 and C6 for the W4‐11 database.

## 4 Discussion

The analysis of EVI in Table 2 and Figures 3, 4, 5 confirmed that local constraints can be violated in semi‐empirical functionals, and to a lesser extent, even in non‐empirical functionals. This observation highlights a potential shortcoming of metrics based on these locally imposed constraints. They might be overly stringent, leading to violations even for functionals that adhere to the global exact condition. In this section, we discuss the EVI metric and ask what does it tell us about the utility of the local constraints of Table 1. Before doing so, we briefly discuss the relationship of the present study to its motivating precedent.

The recent communication by Pederson and Burke [13] introduced the C1‐6 local constraints and looked for violations of the same. Their analysis employed idealized Gedanken densities, contrasting with the molecular densities used in this study. Reference [13] quantified violations by counting the number of violations above certain preset thresholds (Table SII in the Supporting Information). In contrast, EVIs account for the magnitude of the violations, not just their presence. This approach revealed correlations between local constraint violations and errors in total energies. While both metrics offer valuable insights, they serve complementary purposes. A more detailed comparison between these approaches is provided in the section titled “Regarding Evaluation of Local Constraints” in the Supporting Information.

**FIGURE 7:**
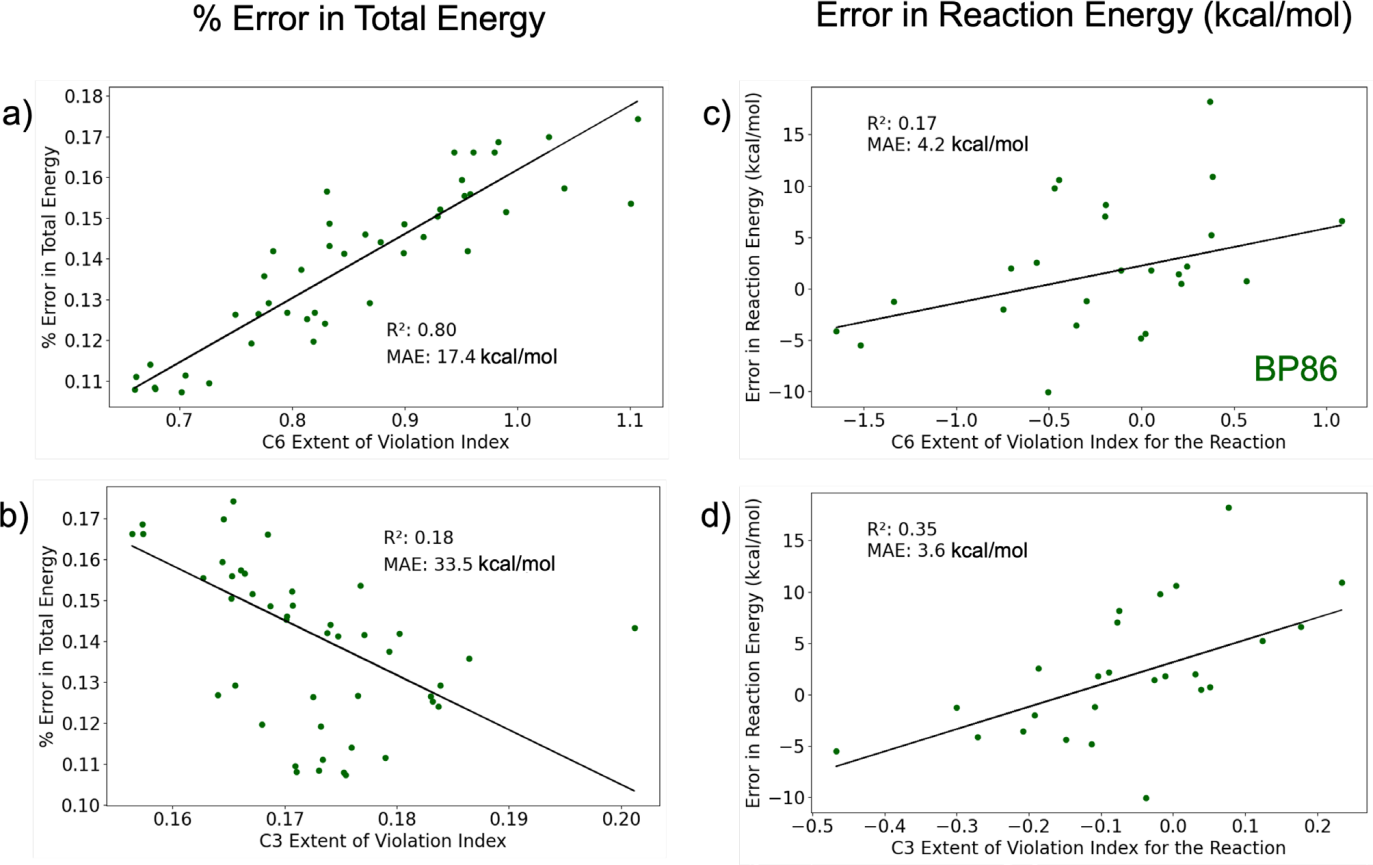
Variation of percent error in total energy with the extent of violation index for (a) local constraint 6 and (b) local constraint 3 for BP86 functional for molecules in the G2RC dataset. Plots (c) and (d) depict the variation of error in reaction energy with the extent of violation index for the reaction for reactions in the G2RC dataset. Mean absolute errors (MAEs) in plots (a) and (b) were converted from percent values to kcal/mol.

Returning to the analysis of local constraints via the present study, the lack of correlation between EVI and atomization energies contrasts with their noticeable relation to total energy for GGA functionals (the latter up to $R^{2}=0.80$, depending on functional and condition). For BP86, where C6 correlated at $R^{2}=0.80$ with the error in total energy, it is natural to ask how this factor carries over into relative energies. Since the atomization energies of Figure 4 are uncorrelated to C6, and also uncorrelated to C1‐5 (Figure S5 in SI), the total energy relationship with EVI appears to have no obvious effect on relative energies. A possible explanation for this is found in Figure 7, which compares the C6 and C3 EVIs to the energy errors in the G2RC dataset. There, the mean absolute errors MAEs (with respect to the linear fit) in total energy are much higher than the MAEs in reaction energy. Even when a substantial variation in total energy is described by a correlation with EVI, there is still a large variation that does not cancel out in a relative energy calculation. While cancellation of errors is undoubtedly present in GGA relative energies [64, 65, 66], it is not easy to pinpoint the source of error cancellation when considering the EVI metrics.

**FIGURE 8:**
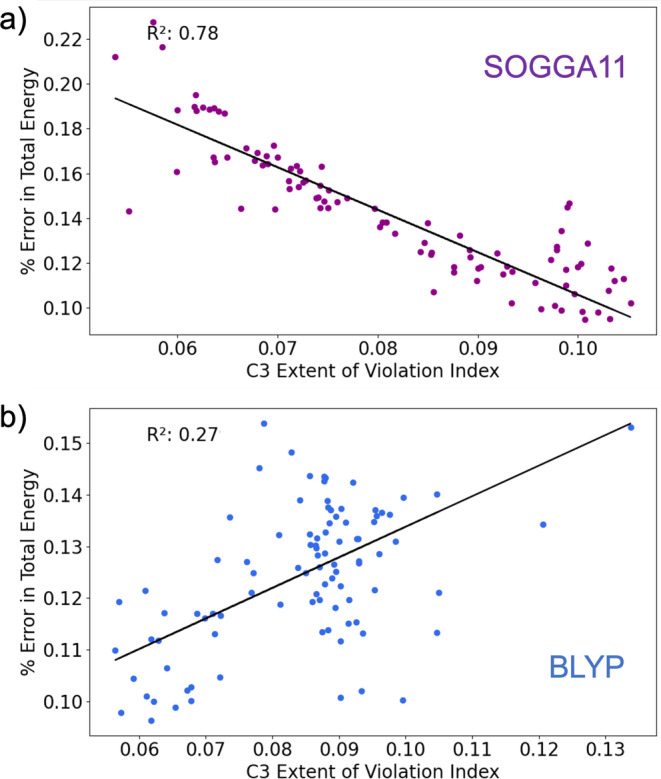
Variation of percent error in total energy with the extent of violation index for local constraint 3 for (a) SOGGA11 and (b) BLYP functionals reported for the W4‐11 database.

The correlation between total energy and EVI can even be negative (Figure 7b), suggesting that increased violation of local constraints can *improve* total energies. While this correlation is weak for BP86 with C3 ($R^{2}=0.18$), a significant $R^{2}$ was found for a related functional. Figure 8 shows relationships between error in total energy and the EVI for C3, particularly for the SOGGA11 and BLYP functionals. These two functionals were chosen because the errors in their total energy predictions showed the strongest negative and positive correlations with the EVI for constraint 3 in the W4‐11 dataset. For the SOGGA11 functional, increased EVI leads to improved total energies, with $R^{2}$ = 0.78.

## 5 Conclusions

This study examined how local constraint violations impact energy computations in DFT through the EVI metric, which quantified these violations for GGA functionals in molecular systems. The metric was applied to a number of GGA exchange‐correlation functionals, showing significant statistical relationships between EVI and total energies for semi‐empirical functionals. Surprisingly, the relationship could even be a negative trend, suggesting a counter‐intuitive possibility: increasing violations for certain local constraints (e.g., C3) might be associated with improved total energies. This result makes clear that local constraints, which are by nature excessive compared to the exact global constraints, can be imposed to the detriment of energetic properties coming from a functional.

## Author Contributions

**Vaibhav Khanna:** conceptualization (equal); data curation (equal); formal analysis (equal); investigation (equal); methodology (equal); software (equal); visualization (equal); writing – original draft (equal); writing – review and editing (equal). **Bikash Kanungo:** conceptualization (supporting); funding acquisition (supporting); validation (supporting); writing – review and editing (supporting). **Vikram Gavini:** conceptualization (supporting); funding acquisition (equal); validation (supporting); writing – review and editing (supporting). **Ambuj Tewari:** conceptualization (supporting); validation (supporting); writing – review and editing (supporting). **Paul M. Zimmerman:** conceptualization (equal); formal analysis (equal); funding acquisition (equal); investigation (equal); project administration (equal); software (equal); supervision (equal); writing – original draft (equal); writing – review and editing (equal).

## Conflicts of Interest

The authors declare no conflicts of interest.

## Supporting information


**Data S1.** Supporting information.

## Data Availability

All source code for the calculations described in this work can be found on our group's GitHub page at https://github.com/ZimmermanGroup/Local_Conditions_DFT.
