# Supplementary material for: Examining the Impact of Local Constraint Violations on Energy Computations in DFT
Source: J Comput Chem. 2025 Jan 2;46(1):e70005. doi: 10.1002/jcc.70005 (PMC11694564; doi:10.1002/jcc.70005)
Supplement: Supplementary file 1 — Data S1. Supporting information. [file JCC-46-0-s001.pdf]

Supporting Information: Examining the Impact of Local Constraint Violations on Energy Computations in DFT

Vaibhav Khanna[1], Bikash Kanungo[2], Vikram Gavini[2,3], Ambuj Tewari[4] and Paul M. Zimmerman[1,*]

[1]*Department of Chemistry, University of Michigan, Ann Arbor, Michigan 48109, United States*
[2]*Department of Mechanical Engineering, University of Michigan, Ann Arbor, Michigan 48109, United States*
[3]*Department of Materials Science & Engineering, University of Michigan, Ann Arbor, Michigan 48109, United States*
[4]*Department of Statistics, University of Michigan, Ann Arbor, Michigan 48109, United States*

October 28, 2024

[1*] *paulzim@umich.edu*

Table of Contents

- Magnitudes of Local Constraints S3
- Regarding Evaluation of Local Constraints S3
- Relative Energy Errors and Violation Indices
  - Atomization Energy S5
  - Reaction Energy S9
  - Ionization Potential S11
- Total Energy Errors and Violation Indices
  - W4-11 Dataset S12
  - G2RC Dataset S16
- Different Choices of Violation Indices S19
- Analytical evaluation of the local constraint for the E_c scaling inequality (C3) for PBE functional S21
- A Simple Model for Constraints S24

Quantity	Minimum Value (a. u.)	Maximum Value (a. u.)
$\epsilon_c[n](\mathbf{r})$	-0.0525	0.0217
F_{xc}	1.0479	490.6259
$\frac{\partial F_c}{\partial r_s}$	-0.5650	0.0880
$\frac{F_c(r_s \to \infty) - F_c}{r_s}$	0.0000	0.3406
$F_{xc} + r_s \frac{\partial F_c}{\partial r_s}$	-0.9474	490.6261
$\frac{\partial}{\partial r_s}\left(r_s^2 \frac{\partial F_c}{\partial r_s} \right)$	-20.2867	0.4328

Table SI: Range of values of quantities that appear in local constraints, computed for He with the BLYP functional.

Magnitudes of Local constraints

Table SI list values of key quantities that appear in the mathematical expressions for local constraints, to give sense of scale for each of these factors. These quantities were computed for He with the BLYP functional.

Regarding Evaluation of Local Constraints

The approach followed by Pederson and Burke[1] to evaluate local constraints searched for potential violations by constructing gedanken densities within realistic ranges for molecules and materials. Violations of the conditions were measured by counting the number of violations against threshold values (specified in Table SII). To compute the derivatives required for C3, C4, C5, and C6, a finite difference approach was used. This means of computing derivatives will be sensitive to the spacing between points. Ref [1] used 10,000 points of r_s over the range [0.0001,5]. We tested this sensitivity by changing the discretization over 7 sets with 10,000 to 500,000 points. This resulted in a change in the results for the E_c scaling local constraint, which was violated for a number of semi-empirical GGA functionals (Table SIII). As the table shows, fewer violations of the local constraints were found with the tighter spacing between points for C3, and no significant changes were found for the other conditions. This analysis suggests it is more precise to use analytical derivatives when evaluating local constraints.

To further analyze the metric of Pederson and Burke, we computed the fraction of grid points where local constraints were violated for molecules in the G2RC dataset. We used the same numerical tolerance values that they used while evaluating local constraints (Table SII). For the BP86 functional, Figure S1 shows how the percent error in total energy varies with the fraction of grid points where local constraints were violated. No correlation was found. However, if we utilise EVI to quantify violations, we see correlation between errors in total energies and EVIs for constraints 2, 3, 5 and 6 (Figure S2). This suggests that EVIs offer a more informative measure to capture the connection between local constraint violations and total energy errors.

#	Exact Condition	Local Constraint Violation Criteria
1	E_c non-positivity[1]	$F_c < -10^{-5}$
2	E_{xc} lower bound[1–3]	$F_{xc} > C_{LO} + 10^{-3}$
3	E_c scaling inequality[1, 4]	$\frac{\partial F_c}{\partial r_s} < -10^{-5}$
4	T_c upper bound[1, 5, 6]	$\frac{\partial F_c}{\partial r_s} r_s - (F_c(r_s \to \infty) - F_c) > 10^{-3}$
5	U_{xc} lower bound[1, 2]	$F_{xc} + r_s \frac{\partial F_c}{\partial r_s} > C_{LO} + 10^{-3}$
6	$U_c(\lambda)$ monotonicity from adiabatic connection[1, 7]	$2\frac{\partial F_c}{\partial r_s} + r_s \frac{\partial^2 F_c}{\partial r_s^2} < -10^{-3}$

Table SII: Criteria for Local Constraint violation used in Ref [1].

Functional	Fraction of points where the E_c scaling inequality local constraint is violated with the following r_s values:						
	10,000	20,000	30,000	40,000	50,000	100,000	500,000
LYP	0.218	0.174	0.144	0.121	0.104	0.050	0.000
P86	0.298	0.176	0.035	0.000	0.000	0.000	0.000
SOGGA11	0.003	0.000	0.000	0.000	0.000	0.000	0.000
GAM	0.459	0.386	0.320	0.260	0.207	0.072	0.000

Table SIII: Sensitivity of the E_c scaling inequality local constraint computation upon changing the number of r_s values in the range [0.0001,5]. Shown are semi-empirical GGAs. Ref [1] used 10,000 points in their study.

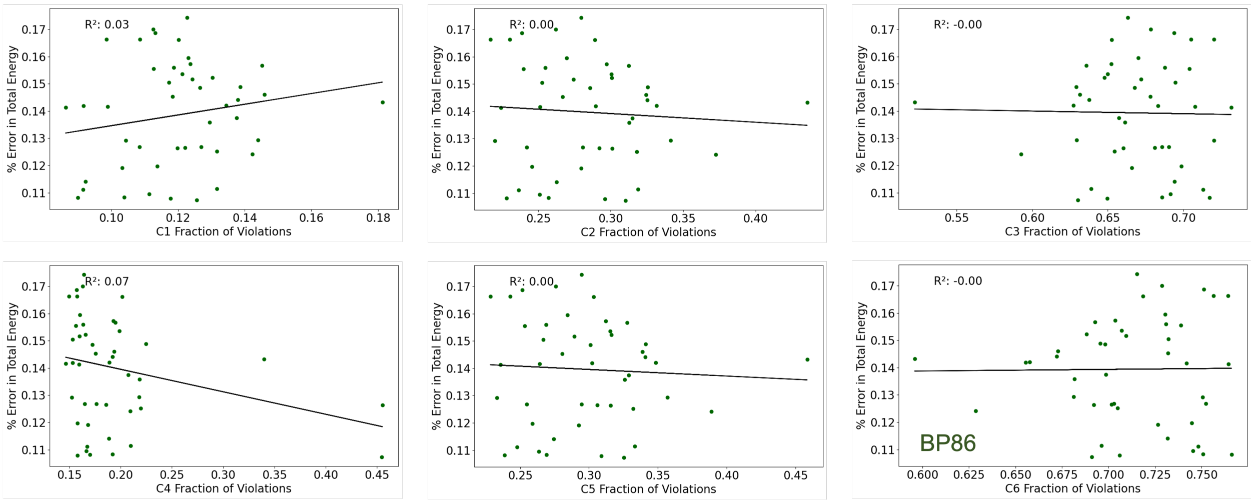

Figure S1: Variation of percent error in total energy with the fraction of grid points where local constraints were violated for BP86 functional reported for the G2RC database (excluding outliers).

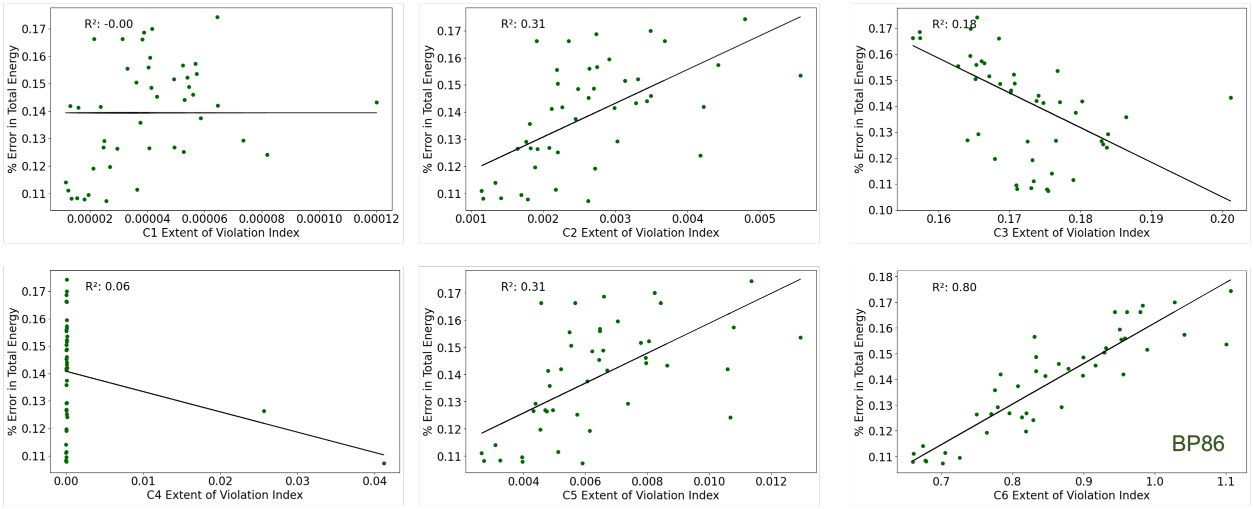

Figure S2: Variation of percent error in total energy with the extent of violation index for local constraints 1 - 6 for BP86 functional reported for the G2RC database (excluding outliers).

Relative Energy Errors and Violation Indices

Atomization Energy

- Figure S3 shows the variation of % error in atomization energy (a, b) and % error in total energy (c, d) with the extent of violation index for local constraint 6 for BP86 functional. Figures b and d exclude molecules H_2 and Be_2 (W4-11 database).

- In our analysis, we selected percent error as the metric for atomization energies to ensure consistency with the total energy analysis, which also uses percent errors. Additionally, because the EVI is normalized across different molecules, it is important to apply a similar normalization to the energy errors. Percent error provides this normalization by assessing errors relative to the magnitude of the atomization energy. This is particularly relevant when dealing with a diverse set of molecules, where larger molecules may inherently have larger absolute errors, which could obscure the relationship between EVI and functional performance. By using percent error, we ensure a fair comparison of how the EVI correlates with functional performance across the dataset. However, we also performed an analysis for BP86 using the absolute error in atomization energy in kcal/mol. As shown in Figure S4, the correlation between the absolute error and EVI remains weak across all six constraints, with the highest R^2 reaching only 0.30 for Constraint 3. This result suggests that using absolute errors does not significantly improve the correlation between energy errors and local constraint violations, and thus, we have chosen to retain percent error for consistency.

- Figures S5 and S6 show no correlation between atomization energy errors and violation indices for BP86 and BLYP functionals (W4-11 dataset, excluding outliers).

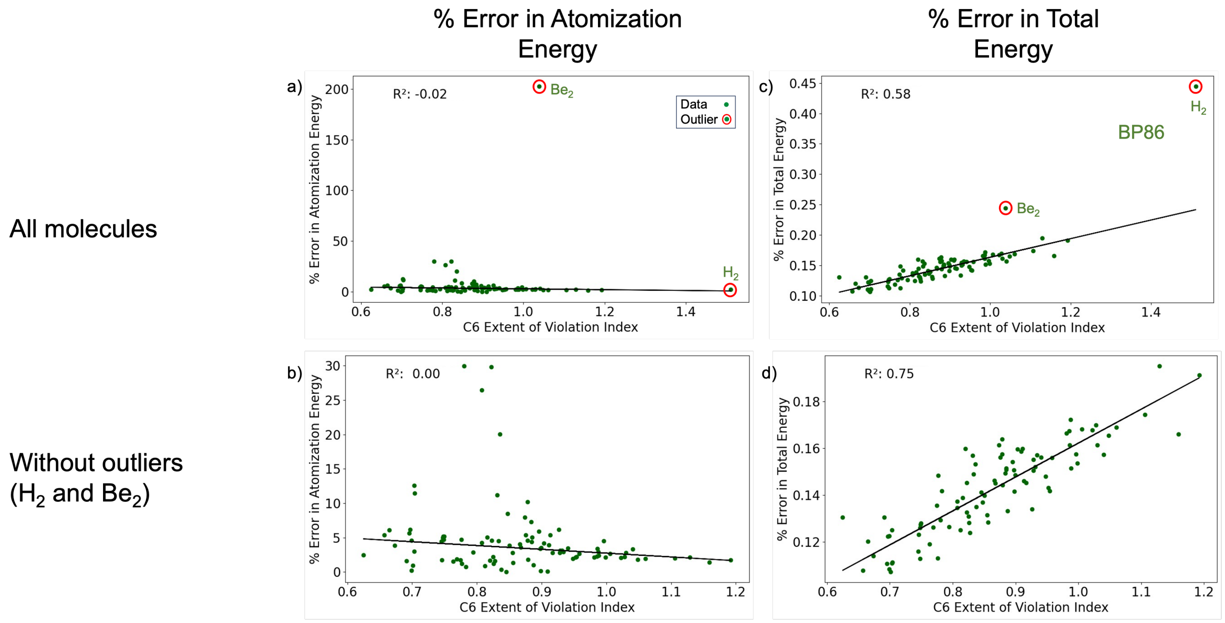

Figure S3: Variation of % error in atomization energy (a, b) and % error in total energy (c, d) with the extent of violation index for local constraint 6 for BP86 functional. Figures b and d exclude molecules H_2 and Be_2 (W4-11 database).

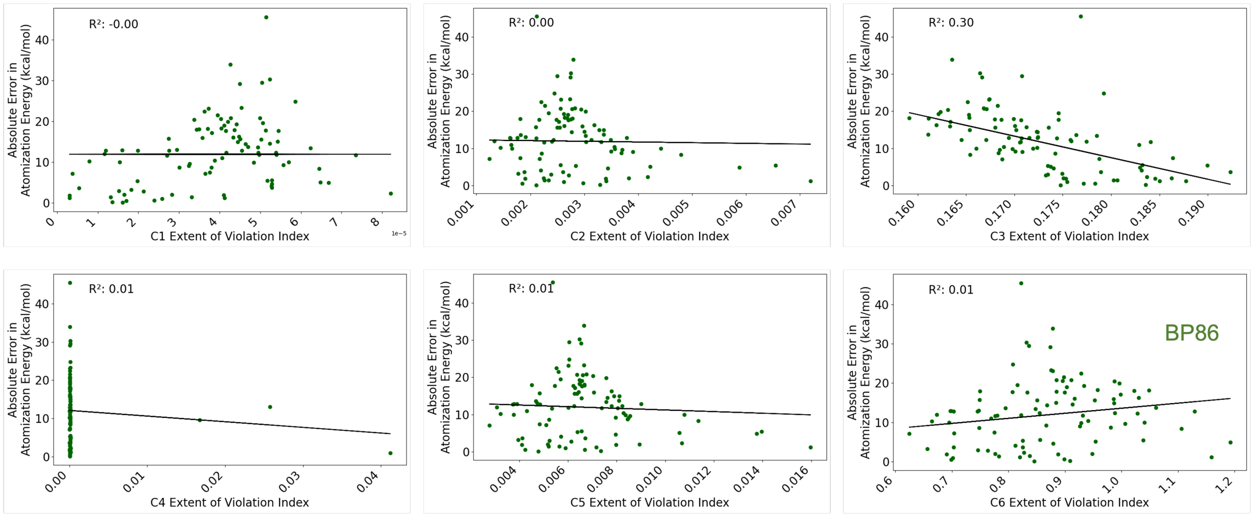

Figure S4: Variation of absolute error in atomization energy (kcal/mol) with the extent of violation index for local constraints 1-6 for BP86 functional (W4-11 database).

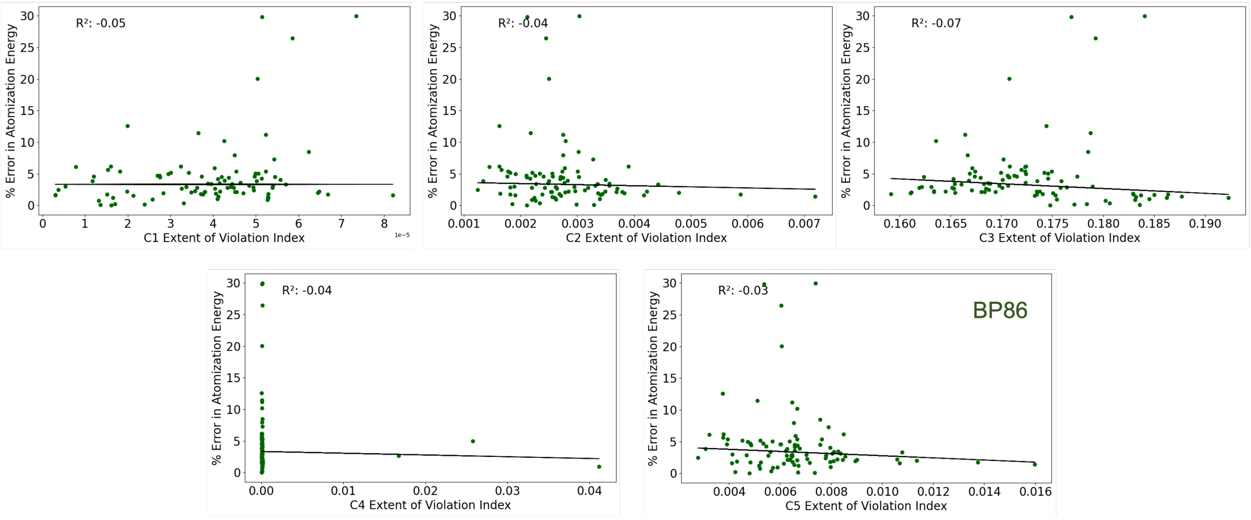

Figure S5: Variation of percent error in atomization energy with the extent of violation index for local constraints 1 - 5 for BP86 functional. (W4-11 database, excluding outliers).

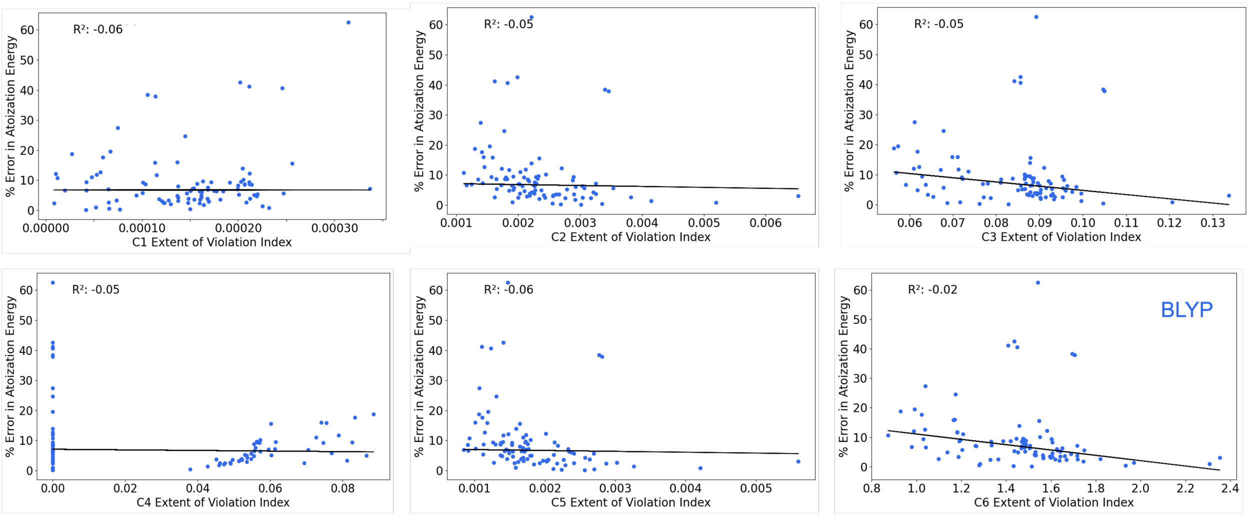

Figure S6: Variation of percent error in atomization energy with the extent of violation index for local constraints 1 - 6 for BLYP functional. (W4-11 database, excluding outliers).

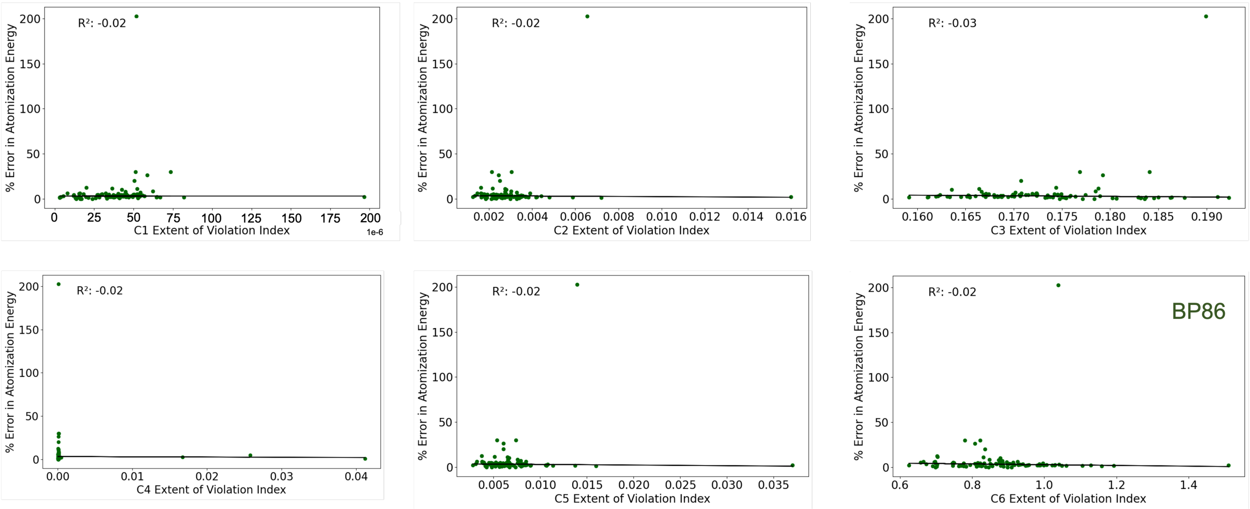

Figure S7: Variation of percent error in atomization energy with the extent of violation index for local constraints 1 - 6 for BP86 functional (W4-11 database, including outliers).

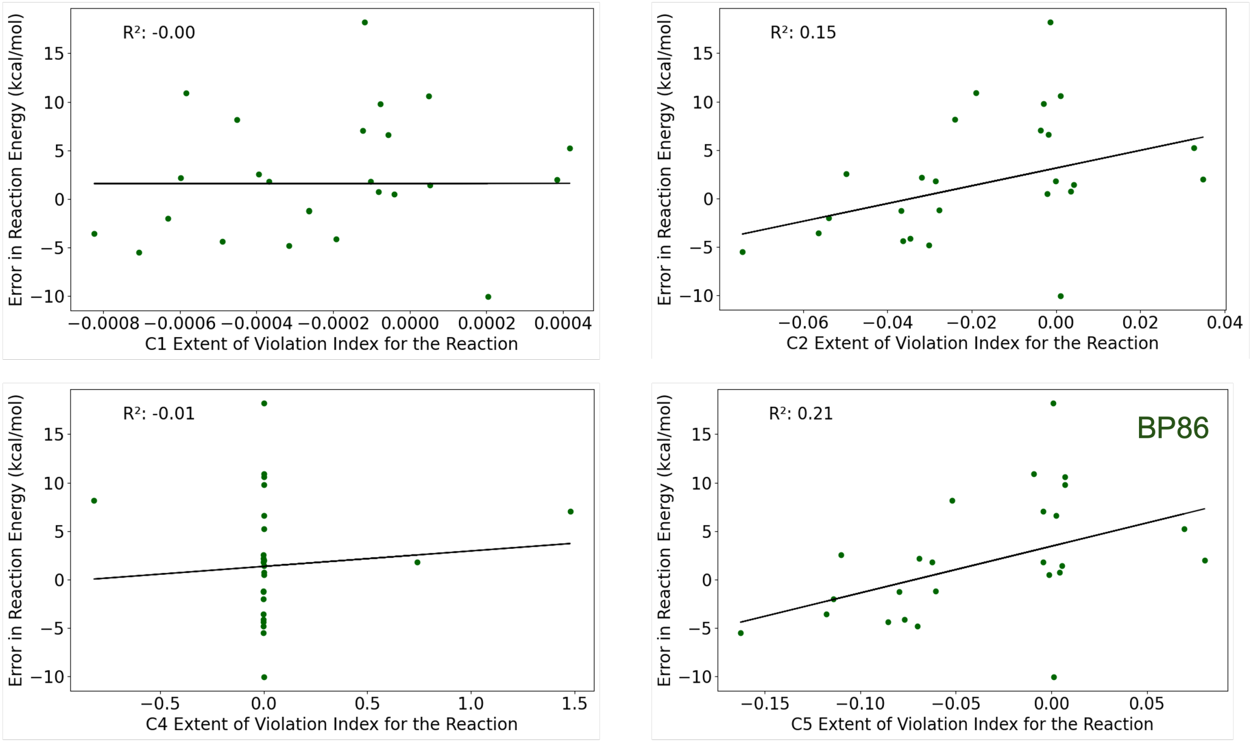

Figure S8: Variation of error in reaction energy with the extent of violation index for the reaction for local constraints 1, 2, 4 and 5 for BP86 functional (G2RC database). The index for the reaction is evaluated as described in the main text.

Reaction Energy

- Figure S8 explores the correlation between BP86 functional's reaction energy errors and violation indices for local constraints 1, 2, 4, and 5 (G2RC database). Some correlation is observed for constraints 2 and 5.

- Figure S9 investigates the correlation for all local constraints (G2RC database) with the same functional. The index for the reaction is now computed differently, evaluated without multiplying individual molecular EVIs with their number of electrons. Except for condition 3, there is no correlation between errors and violation indices.

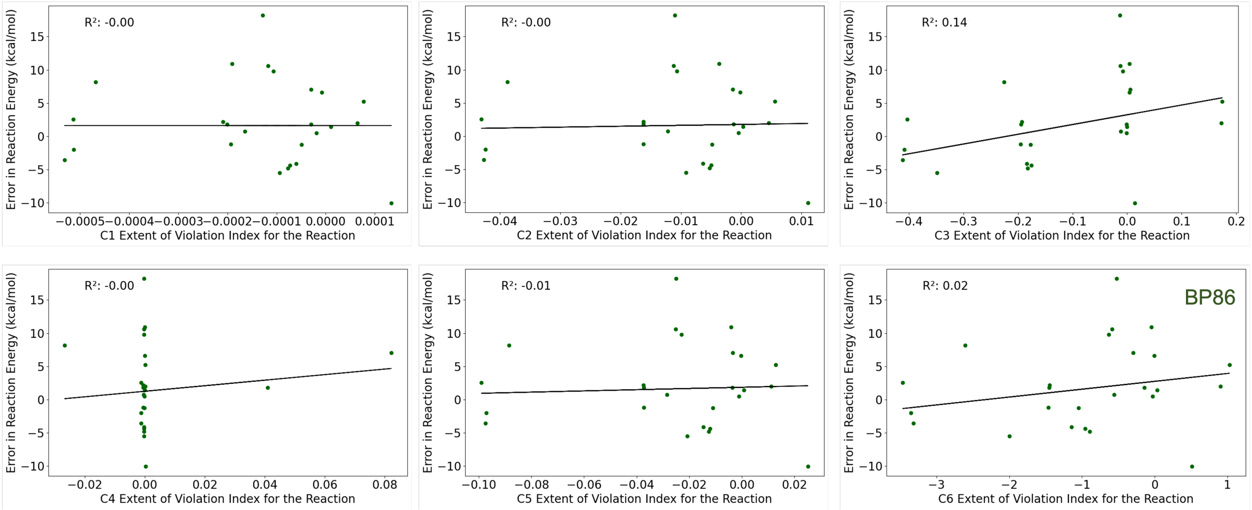

Figure S9: Variation of error in reaction energy with the extent of violation index for the reaction for all local constraints for BP86 functional (G2RC database). The index for the reaction is evaluated without multiplying individual EVIs with number of electrons.

Ionization Potential

- Figure S10 explores the correlation between BP86 functional's ionization potential errors and violation indices for local constraints 1-6 for neutral, closed shell species from the G21IP database [8]. There is no correlation between percent error in ionization potential and violation indices.

- Figure S11 depicts the variation of ionization potential errors and violation indices for local constraints 3, 4 and 6 for neutral, closed-shell species from the G21IP database [8] for PBE. There is no correlation between percent error in ionization potential and violation indices. The violation indices for constraints 1, 2, and 5 were zero, hence not plotted.

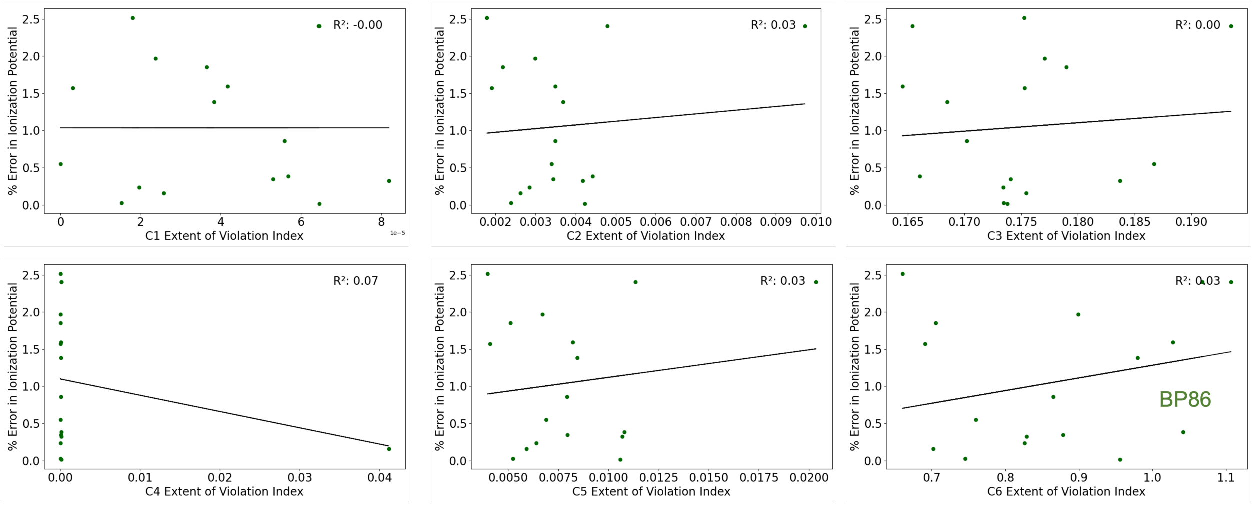

Figure S10: Variation of percent error in ionization potential with the extent of violation index for local constraints 1 - 6 for BP86 functional reported for the G21IP database.

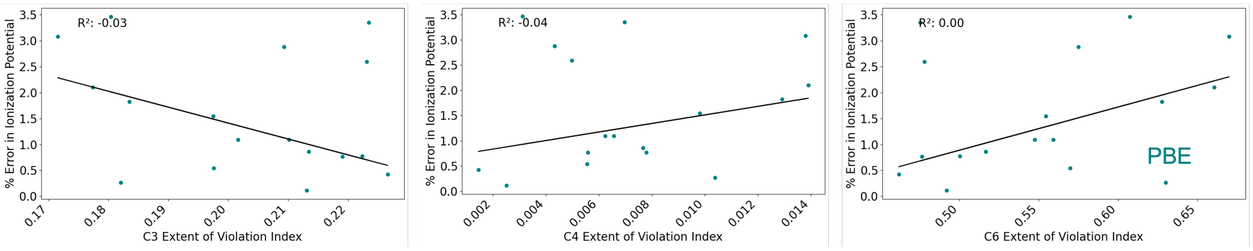

Figure S11: Variation of percent error in ionization potential with the extent of violation index for local constraints 3, 4 and 6 for PBE functional reported for the G21IP database.

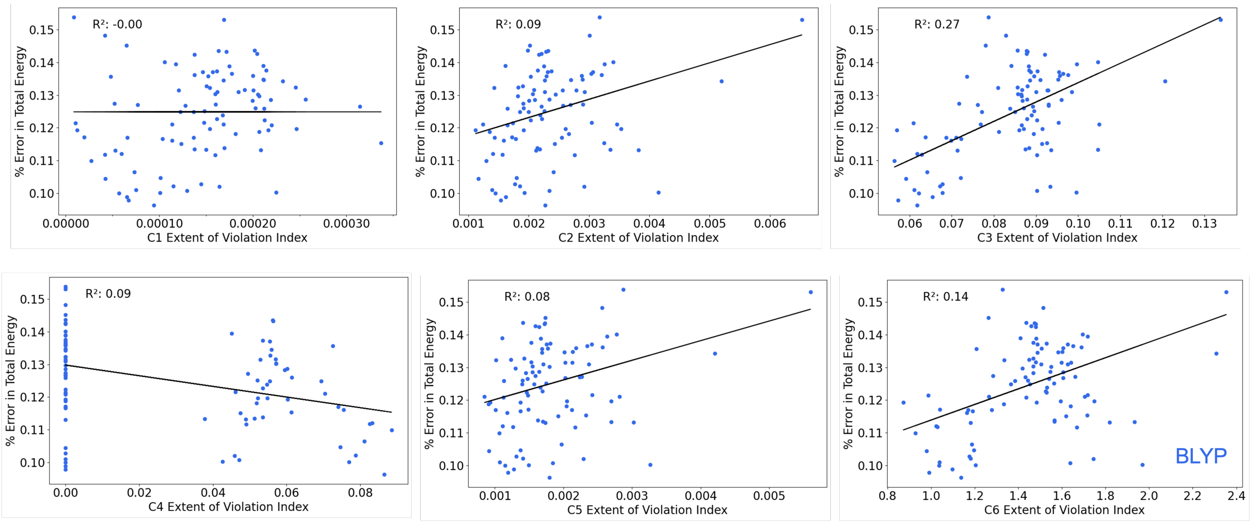

Figure S12: Variation of percent error in total energy with the extent of violation index for local constraints 1 - 6 for BLYP functional reported for the W4-11 database (excluding outliers).

Total Energy Errors and Violation Indices

W4-11 Dataset

Figures S12, S13, S14, S15, S16 and S17 analyze the variation of percent error in total energy with local constraint violation indices for semi-empirical functionals for closed-shell neutral molecules in the W4-11 database (excluding outliers).

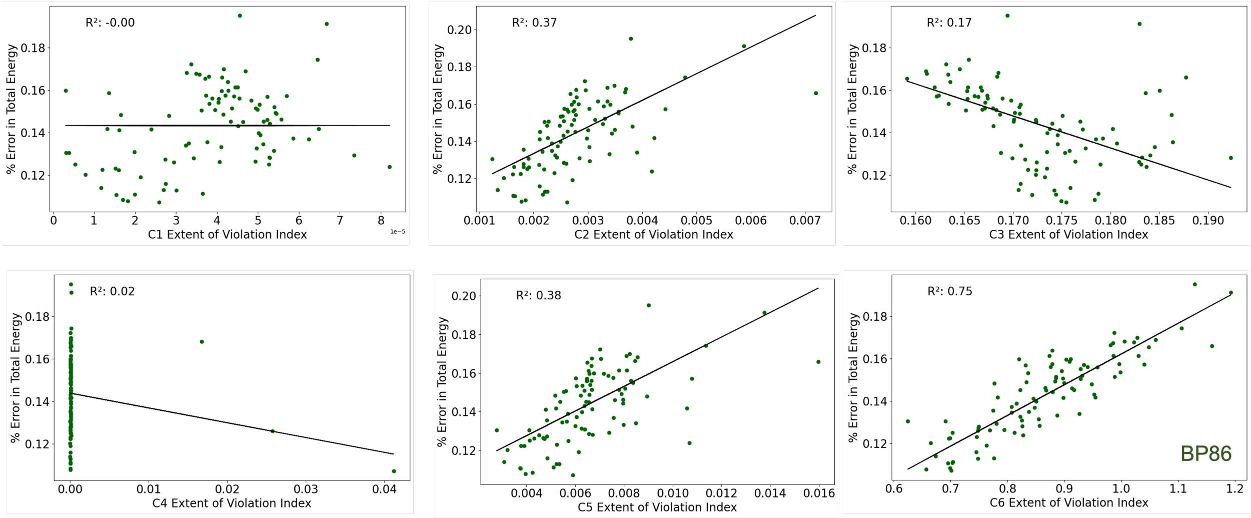

Figure S13: Variation of percent error in total energy with the extent of violation index for local constraints 1 - 6 for BP86 functional reported for the W4-11 database (excluding outliers).

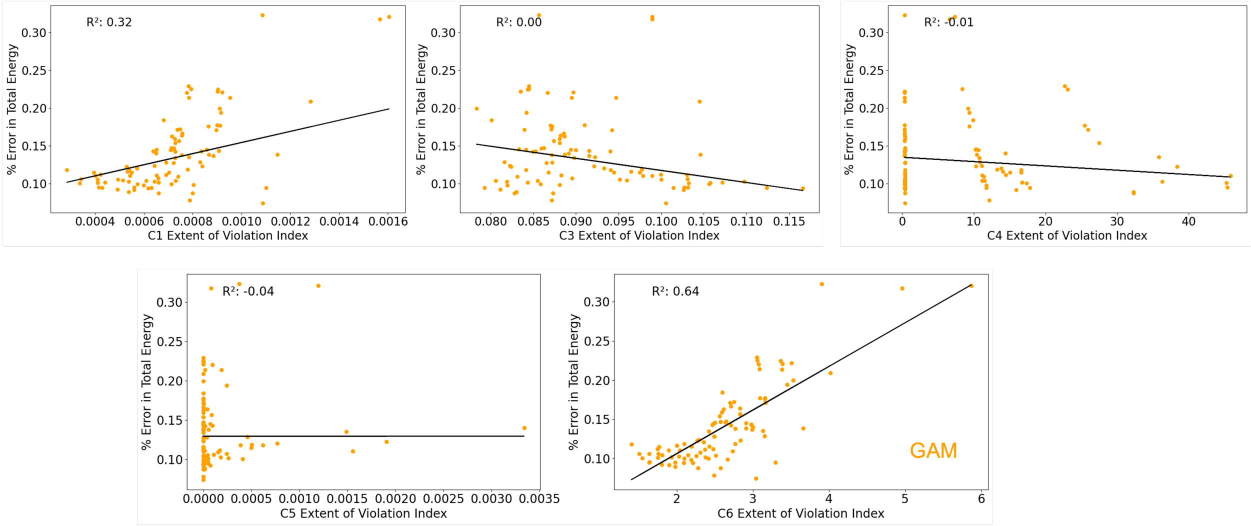

Figure S14: Variation of percent error in total energy with the extent of violation index for local constraints 1, 3, 4, 5 and 6 for GAM functional reported for the W4-11 database (excluding outliers).

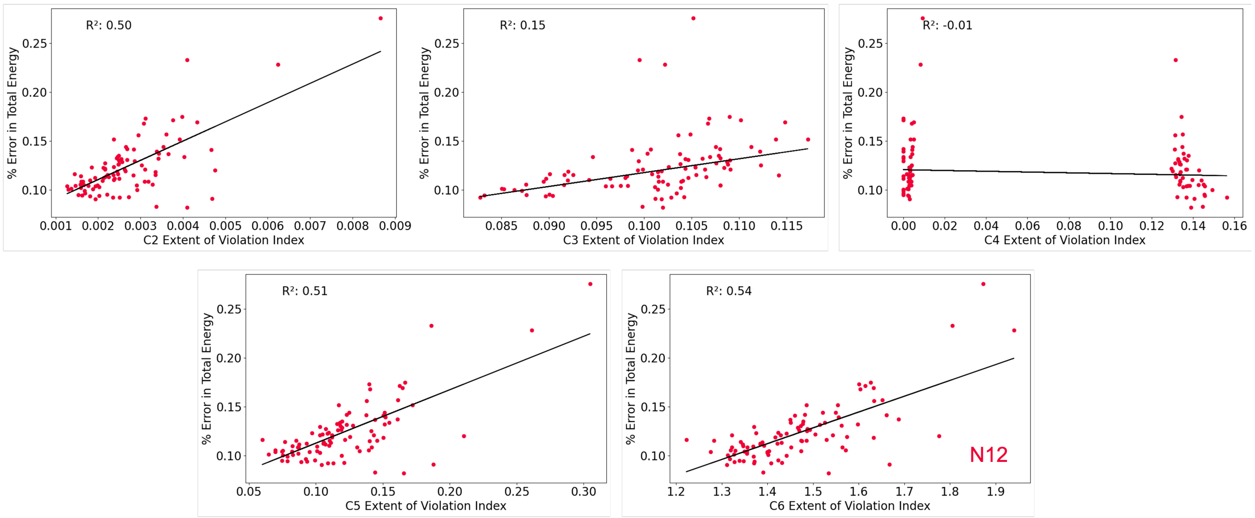

Figure S15: Variation of percent error in total energy with the extent of violation index for local constraints 2 - 6 for N12 functional reported for the W4-11 database (excluding outliers).

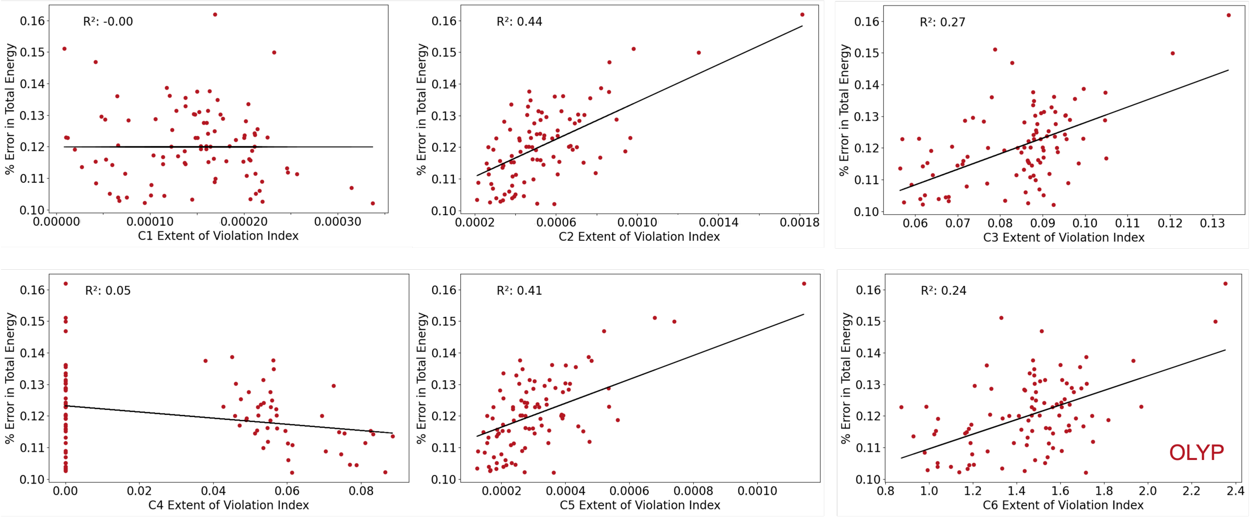

Figure S16: Variation of percent error in total energy with the extent of violation index for local constraints 1 - 6 for OLYP functional reported for the W4-11 database (excluding outliers).

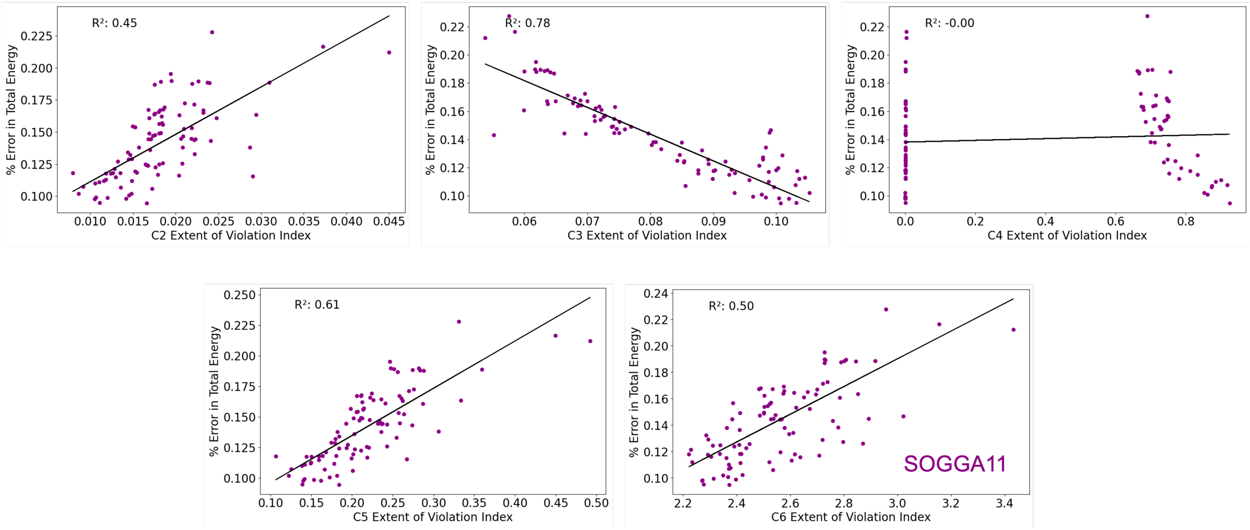

Figure S17: Variation of percent error in total energy with the extent of violation index for local constraints 2 - 6 for SOGGA11 functional reported for the W4-11 database (excluding outliers).

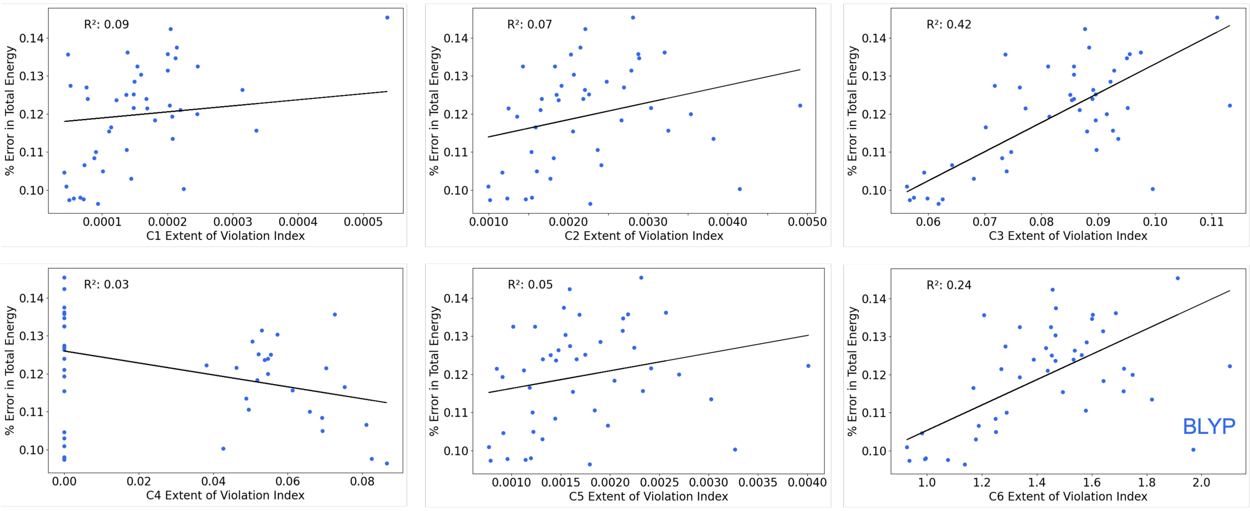

Figure S18: Variation of percent error in total energy with the extent of violation index for local constraints 1 - 6 for BLYP functional reported for the G2RC database (excluding outliers).

G2RC Dataset

Figures S18, S2, S19, S20, S21 and S22 examine the variation of percent error in total energy with local constraint violation indices for closed-shell neutral molecules in the G2RC database (excluding outliers).

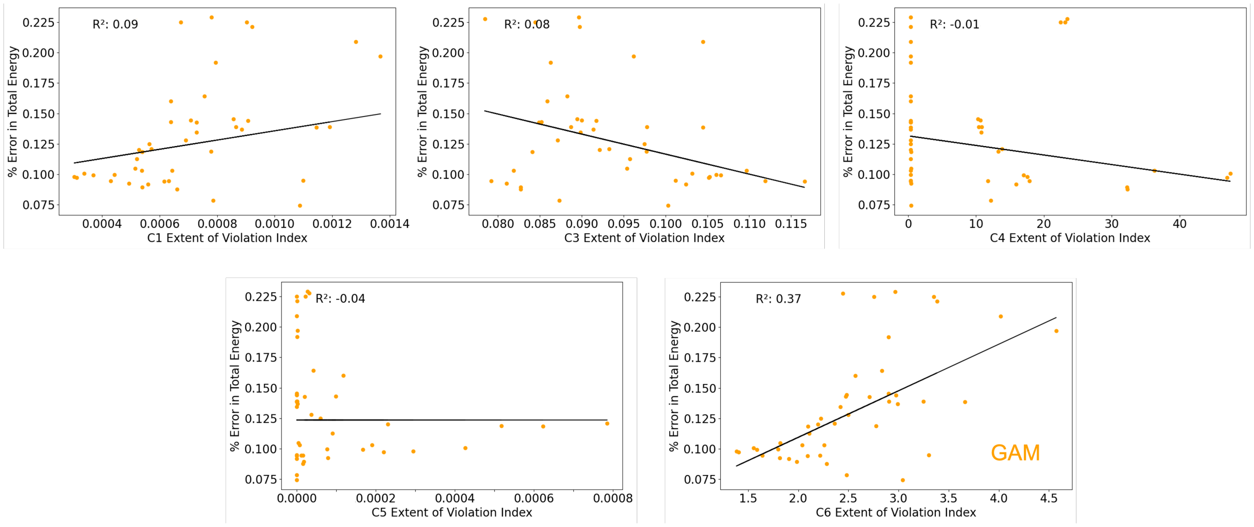

Figure S19: Variation of percent error in total energy with the extent of violation index for local constraints 1, 3, 4, 5 and 6 for GAM functional reported for the G2RC database (excluding outliers).

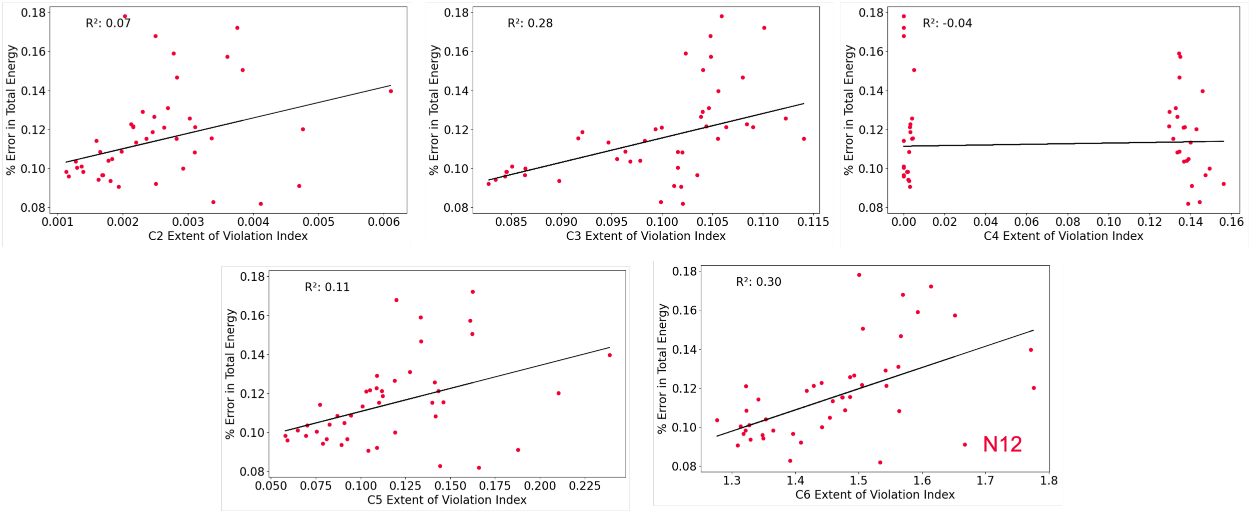

Figure S20: Variation of percent error in total energy with the extent of violation index for local constraints 2 - 6 for N12 functional reported for the G2RC database (excluding outliers).

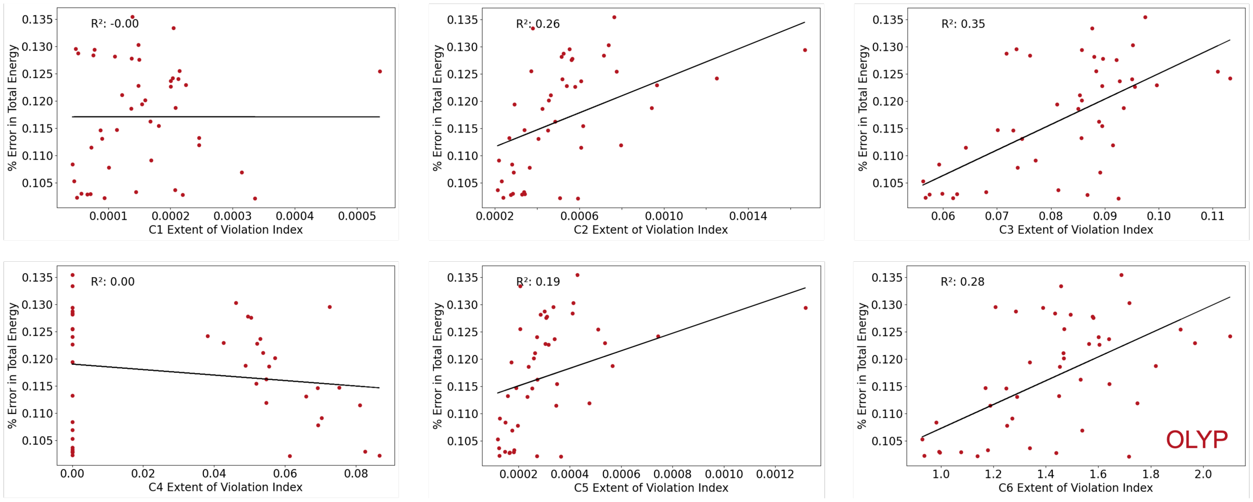

Figure S21: Variation of percent error in total energy with the extent of violation index for local constraints 1 - 6 for OLYP functional reported for the G2RC database (excluding outliers).

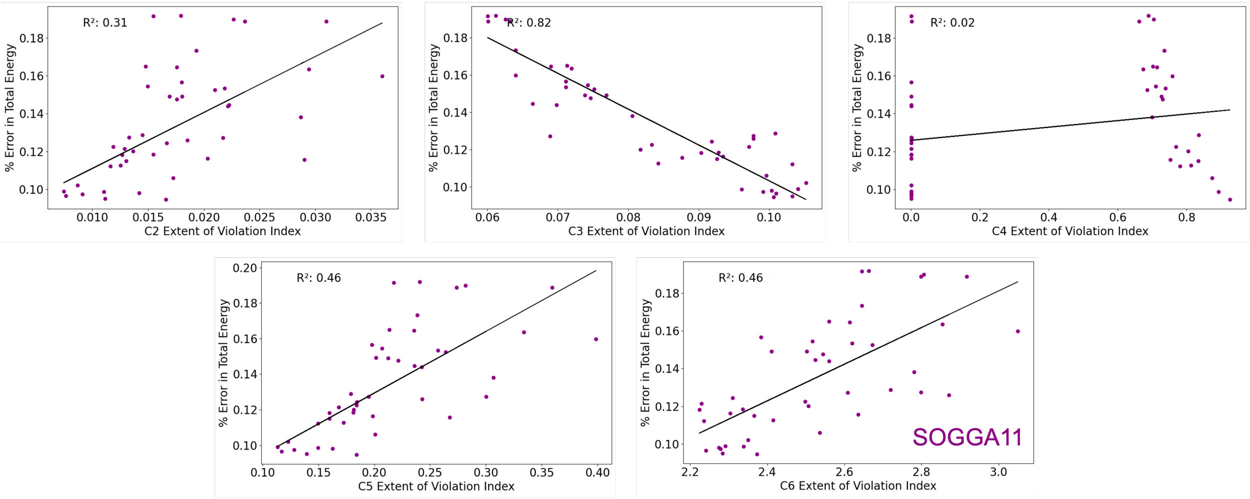

Figure S22: Variation of percent error in total energy with the extent of violation index for local constraints 2 - 6 for SOGGA11 functional reported for the G2RC database (excluding outliers).

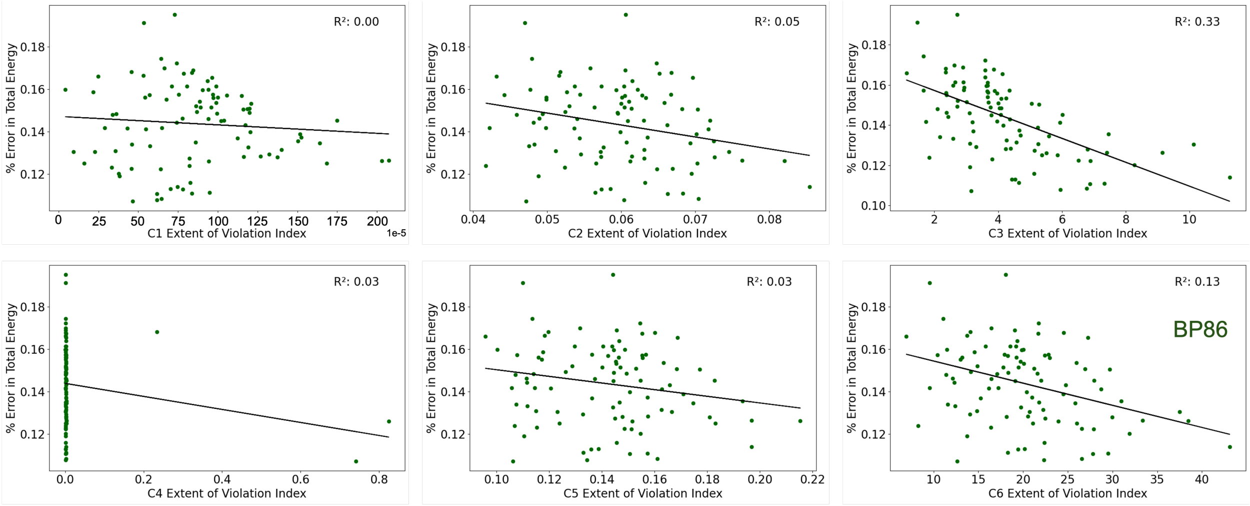

Figure S23: Variation of percent error in total energy with the extent of violation index for local constraints 1 - 6 for BP86 functional reported for the W4-11 database (excluding outliers). EVI values were not divided by the number of electrons.

Different Choices of Violation Indices

In the main text, the extent of violation index was defined as follows:

$$EVI = \frac{\int g(\mathbf{r})n(\mathbf{r})d\mathbf{r}}{\int n(\mathbf{r})d\mathbf{r}} \tag{1}$$

$$g(\mathbf{r}) = \begin{cases} |violation| & \text{if local constraint is violated} \\ 0 & \text{otherwise} \end{cases} \tag{2}$$

We also computed the EVI without dividing by the number of electrons N = $\int n(\mathbf{r})d\mathbf{r}$.

$$EVI = \int g(\mathbf{r})n(\mathbf{r})d\mathbf{r} \tag{3}$$

Figure S23 shows the variation of percent error in total energy with the extent of violation index for local constraints 1 - 6 for BP86 functional reported for the W4-11 database. EVI values in this figure were computed as per equation 3.

Furthermore, instead of weighting violations by the electron density, we recomputed EVI by weighting with the exchange energy density for an unpolarized uniform electron gas ϵ_x^{unif} = $-(3/4\pi)(3\pi^2 n)^{1/3}$.

$$EVI = \int g(\mathbf{r})\epsilon_x^{unif}(\mathbf{r})d\mathbf{r} \tag{4}$$

Figure S24 again shows the variation of percent error in total energy with the extent of violation index for local constraints 1 - 6 for BP86 functional reported for the W4-11 database. EVI values in this figure were computed as per equation 4.

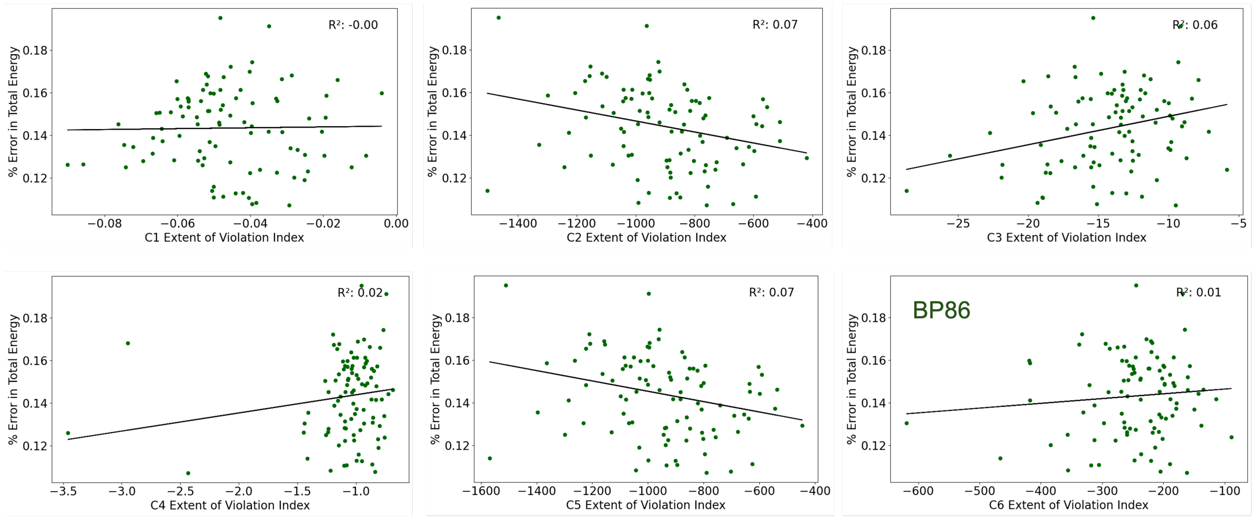

Figure S24: Variation of percent error in total energy with the extent of violation index for local constraints 1 - 6 for BP86 functional reported for the W4-11 database (excluding outliers). Violations are weighted by exchange energy density and EVI values are not divided by the number of electrons.

With these new definitions of EVI, we lost the strong statistical correlation between the % error in total energy and the EVI for local constraint 6 that was observed with the original EVI for BP86.

Analytical evaluation of the local constraint for the E_c scaling inequality (C3) for PBE functional

The correlation energy density for PBE is given as [9]:

$$\epsilon_c^{PBE} = \epsilon_c^{PW92}(r_s,\zeta) + H(r_s,\zeta,t) \tag{5}$$

where ϵ_c^{PW92} is the correlation energy density for the PW92 functional [10], t = $|\nabla n|/2\phi k_s n$, $\phi(\zeta) = [(1+\zeta)^{2/3} + (1-\zeta)^{2/3}]/2$, $n = 3/4\pi r_s^3 = k_F^3/3\pi^2$, $k_s = (4k_F/\pi a_0)^{1/2}$, $a_0 = \hbar^2/me^2$, $\beta \approx 0.066725$, $\gamma \approx 0.031091$.

$$H = \frac{e^2\gamma\phi^3}{a_0}\ln\left(1 + \frac{\beta}{\gamma}t^2\left[\frac{1+At^2}{1+At^2+A^2t^4}\right]\right) \tag{6}$$

$$A = \frac{\beta}{\gamma}\left[\exp\left(\frac{-\epsilon_c^{PW92}}{\gamma\phi^3 e^2/a_0}\right) - 1\right]^{-1} \tag{7}$$

In atomic units, $a_0 = \hbar$ = e = m = 1. For unpolarized densities, $\phi = 1$.

Let

$$t^2\left[\frac{1+At^2}{1+At^2+A^2t^4}\right] = x \tag{8}$$

Then,

$$H = \gamma\ln\left[1 + \frac{\beta}{\gamma}x\right] \tag{9}$$

$$A = \frac{\beta}{\gamma}\left[\exp\left(\frac{-\epsilon_c^{PW92}}{\gamma}\right) - 1\right]^{-1} \tag{10}$$

From Ref [1] and our own work, we know that PW92 satisfies C3. Figure S25 shows $\frac{\partial F_C^{PW92}}{\partial r_s}$ plotted out for H_2 along the bond axis; clearly $\frac{\partial F_C^{PW92}}{\partial r_s} \geq 0$. We then need to check the behaviour of $\frac{\partial}{\partial r_s}(\frac{H}{\epsilon_x^{unif}})$, which now follows.

$$\frac{\partial}{\partial r_s}\left(\frac{H}{\epsilon_x^{unif}}\right) = \frac{\frac{\partial}{\partial r_s}H}{\epsilon_x^{unif}} - \frac{(\frac{\partial}{\partial r_s}\epsilon_x^{unif})H}{(\epsilon_x^{unif})^2} \tag{11}$$

$$\frac{\partial H}{\partial r_s} = \frac{\beta}{1+\beta x/\gamma}\left[\left(\frac{\partial x}{\partial A}\frac{\partial A}{\partial \epsilon_c^{PW92}}\frac{\partial \epsilon_c^{PW92}}{\partial r_s}\right) + \left(\frac{\partial x}{\partial t}\frac{\partial t}{\partial r_s}\right)\right] \tag{12}$$

$$\frac{\partial x}{\partial A} = -\frac{At^6(At^2+2)}{(A^2t^4+At^2+1)^2} \tag{13}$$

$$\frac{\partial A}{\partial \epsilon_c^{PW92}} = \frac{\beta\exp(\frac{-\epsilon_c^{PW92}}{\gamma})}{\gamma^2(\exp(\frac{-\epsilon_c^{PW92}}{\gamma}) - 1)^2} \tag{14}$$

$$\frac{\partial x}{\partial t}\frac{\partial t}{\partial r_s} = \frac{7g^2b^4r_s^6(b^2+2g^2Ar_s^7)}{(b^4+g^2Ab^2r_s^7+g^4A^2r_s^{14})^2} \tag{15}$$

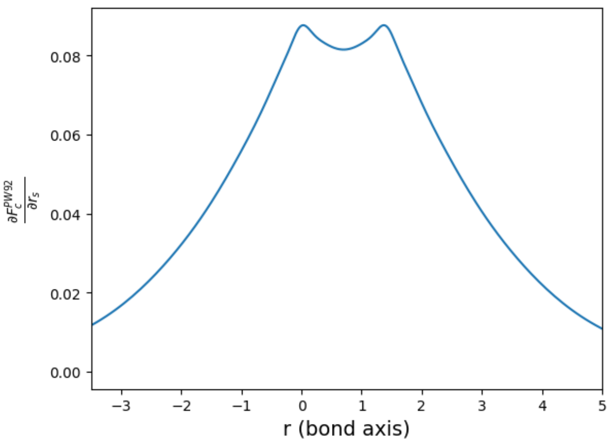

Figure S25: $\frac{\partial F_C^{PW92}}{\partial r_s}$ for H_2 along the bond axis.

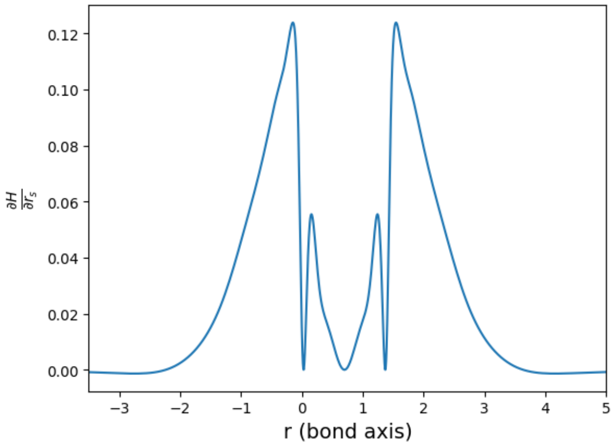

Figure S26: $\frac{\partial H}{\partial r_s}$ plotted out for H_2 along the bond axis.

$$g = |\nabla n|, b = 2\,(4/\pi)^{1/2}\left((9\pi/4)^{1/3}\right)^{1/2}(3/4\pi) \tag{16}$$

Substituting equations 13, 14, 15 into equation 12, and taking $\frac{\partial \epsilon_c^{PW92}}{\partial r_s}$ from LibXC [11], we find that $\frac{\partial H}{\partial r_s} \geq 0$ for H_2 (Figure S26), which then leads to negative values of $\frac{\partial}{\partial r_s}(\frac{H}{\epsilon_x^{unif}})$ (Figure S27).

We then compute $\frac{\partial F_C^{PBE}}{\partial r_s}$ as follows:

$$\frac{\partial F_C^{PBE}}{\partial r_s} = \frac{\partial F_C^{PW92}}{\partial r_s} + \frac{\partial}{\partial r_s}\left(\frac{H}{\epsilon_x^{unif}}\right) \tag{17}$$

For H_2, Figure S28 shows the plot of $\frac{\partial F_C^{PBE}}{\partial r_s}$ along the bond axis. Regions where $\frac{\partial F_C^{PBE}}{\partial r_s} < 0$ are apparent. Hence, there are regions of space where PBE violates the local constraint for the E_c scaling inequality (C3).

Appendix H in Ref [1] evaluates the same condition for the PBE functional. However, their quotient rule is incorrect in equation H5 (c.f. equation 11) due to an incorrect sign on

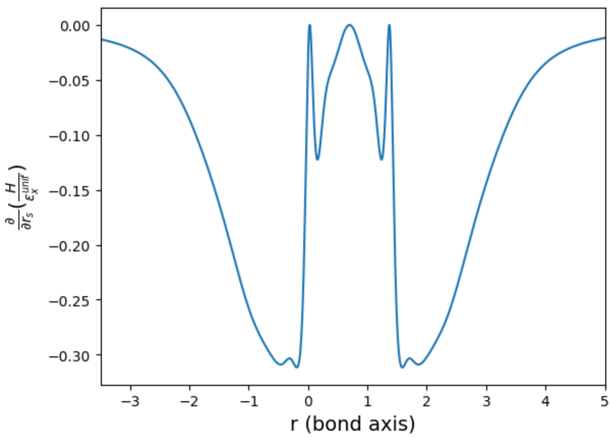

Figure S27: $\frac{\partial}{\partial r_s}(\frac{H}{\epsilon_x^{unif}})$ plotted out for H_2 along the bond axis.

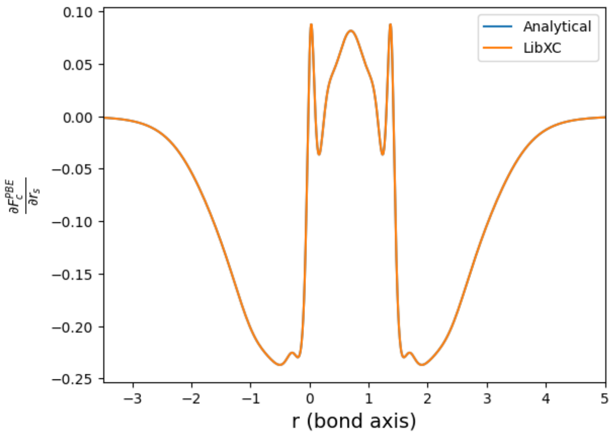

Figure S28: $\frac{\partial F_C^{PBE}}{\partial r_s}$ plotted out for H_2 along the bond axis, computed analytically and through LibXC. The curves are on top of each other.

the second term. Also, their expansion of $\frac{\partial H}{\partial r_s}$ (equation H6 in Ref [1]) skips the derivatives of t, which depends on r_s. We account for this in equation 12. These differences are sufficient to arrive at different conclusions about whether PBE violates the C3 constraint, taking H_2 as a numerical example.

The derivative $\frac{\partial F_C^{PBE}}{\partial r_s}$ computed here matches exactly with the derivative obtained from LibXC, where the latter procedure gave the results of the main paper (Figure S28).

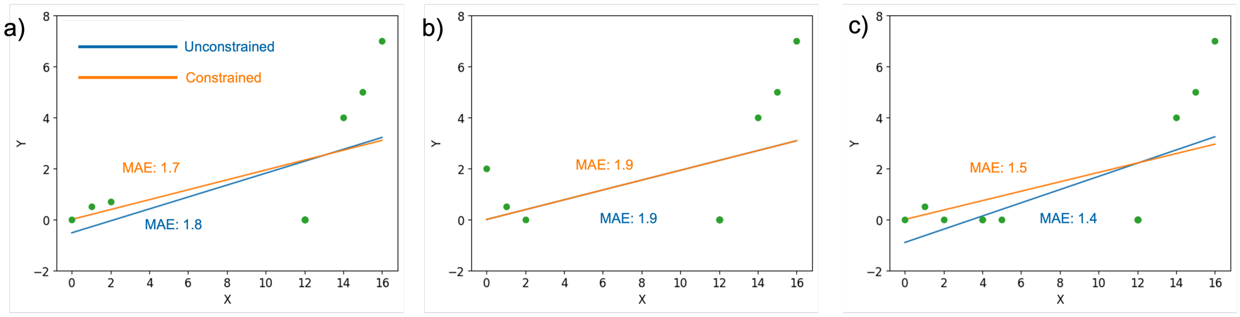

Figure S29: Regression experiment where a linear model was fit to predict a non-negative target based on one feature. Enforcing the non-negativity constraint led to a diverse range of outcomes: a) improved predictions, b) no significant change and c) slightly worse predictions, as measured by the mean absolute error (MAE).

A Simple Model for Constraints

We found the statistical relationships of Figure 7b and Figure 8a to be counterintuitive. The same kind of behavior is possible even in very simple settings, and is not unique to exchange-correlation functionals. Figure S29 compares linear regression models ($y = mx + b$) for three datasets where $y \geq 0$. These datasets are entirely invented, but represent a prototypical case where a dataset is to be fit to a simple, low-dimensional model. The linear fit can be done with or without enforcing the constraint $y \geq 0$. As can been seen, all three possibilities emerge: (a) the constrained model has lower MAE, even though the unconstrained model is more accurate for points where constraints are violated (see Figure S30), (b) the model is the same with or without constraints, and (c) the model has higher MAE upon application of the constraint. Depending on the quality of the training data, the quality of the model, and the form of the constraint, the constrained models may or may not perform better than the baseline.

How does this simple model fit into the context of semilocal density functional development? Imagine proposing a density functional form without any explicit constraints. Training such a form on a database of molecular properties would result in an empirical functional. Alternatively, one can impose constraints (or less rigidly, penalties proportional to constraint violation) upon these forms during the parameterization process. In the linear regression of Figure S29, it is easy to see how improvements in one region can lead to worse modeling in another. While the underlying equations defining the functional would remain the same, the resulting functionals with constraints may or may not improve compared to the less constrained functional. Therefore as in the studies using the EVI metric, the use of constraints does not guarantee improvements in accuracy.

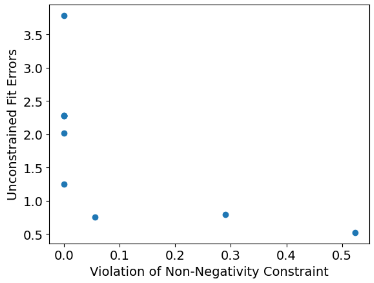

Figure S30: Plot depicting the variation of errors in the unconstrained model fit in Figure S29 (a) with the violation of y non-negativity constraint.

References

(1) Pederson, R.; Burke, K. *J. Chem. Phys.* **2023**, *159*, 214113.

(2) Lieb, E. H. *Int. J. Quantum Chem.* **1983**, *24*, 243–277.

(3) Ziesche, P.; Eschrig, H. In 1991.

(4) Levy, M.; Perdew, J. P. *Phys. Rev. A* **1985**, *32*, 2010–2021.

(5) Levy, M.; Perdew, J. P. *Int. J. Quantum Chem.* **1994**, *49*, 539–548.

(6) Levy, M. *Phys. Rev. A* **1991**, *43*, 4637–4646.

(7) Levy, M.; Perdew, J. P. *Phys. Rev. B* **1993**, *48*, 11638–11645.

(8) Goerigk, L.; Grimme, S. *J. Chem. Theory Comput.* **2010**, *6*, 107–126.

(9) Perdew, J. P.; Burke, K.; Ernzerhof, M. *Phys. Rev. Lett.* **1996**, *77*, 3865–3868.

(10) Perdew, J. P.; Wang, Y. *Phys. Rev. B* **1992**, *45*, 13244–13249.

(11) Lehtola, S.; Steigemann, C.; Oliveira, M. J.; Marques, M. A. *SoftwareX* **2018**, *7*, 1–5.
